# Silicon and Iron as Resource-Efficient Anode Materials for Ambient-Temperature Metal-Air Batteries: A Review

**DOI:** 10.3390/ma12132134

**Published:** 2019-07-02

**Authors:** Henning Weinrich, Yasin Emre Durmus, Hermann Tempel, Hans Kungl, Rüdiger-A. Eichel

**Affiliations:** 1 Institute of Energy and Climate Research-Fundamental Electrochemistry (IEK-9), Forschungszentrum Jülich GmbH, 52428 Jülich, Germany; 2 RWTH Aachen University, Institute of Physical Chemistry, Landoltweg 2, 52074 Aachen, Germany

**Keywords:** aqueous electrolyte, corrosion, iron-air, metal-air batteries, silicon-air, stationary energy storage

## Abstract

Metal-air batteries provide a most promising battery technology given their outstanding potential energy densities, which are desirable for both stationary and mobile applications in a “beyond lithium-ion” battery market. Silicon- and iron-air batteries underwent less research and development compared to lithium- and zinc-air batteries. Nevertheless, in the recent past, the two also-ran battery systems made considerable progress and attracted rising research interest due to the excellent resource-efficiency of silicon and iron. Silicon and iron are among the top five of the most abundant elements in the Earth’s crust, which ensures almost infinite material supply of the anode materials, even for large scale applications. Furthermore, primary silicon-air batteries are set to provide one of the highest energy densities among all types of batteries, while iron-air batteries are frequently considered as a highly rechargeable system with decent performance characteristics. Considering fundamental aspects for the anode materials, i.e., the metal electrodes, in this review we will first outline the challenges, which explicitly apply to silicon- and iron-air batteries and prevented them from a broad implementation so far. Afterwards, we provide an extensive literature survey regarding state-of-the-art experimental approaches, which are set to resolve the aforementioned challenges and might enable the introduction of silicon- and iron-air batteries into the battery market in the future.

## 1. Introduction

Climate change is, beyond any doubt, one of the most important albeit abstract threats of our time. The Earth’s climate is changing due to the action of mankind and requires a great endeavor to be preserved. Restricting global warming to 2 °C till the end of the 21st century, if still possible at all, is a challenge of a generation that has to be engaged on a global scale [1,2,3,4]. In this sense, particularly the emission of greenhouse gases like CO_2_ has to be reduced, but must not impair the continuous power supply to the global population in order to ensure public and economic acceptance [5,6]. Neglecting nuclear options due to safety and waste disposal issues, at least in Germany the reduction of CO_2_ emission necessitates the broad implementation of mostly intermittent renewable energy sources into the electric grid [7,8]. However, this will only be successful by the creation of smart electric grids and backup power supply in a highly renewable energy supply scenario (>80% renewables) [9,10]. Apart from long-term energy transformation approaches, commonly summarized as ‘Power-to-X’ concepts (days–months) [11], continuous short-term electricity supply (hours–days) may be provided by electrochemical energy storage facilities like batteries, which are also extremely important for mobile applications and have been under investigation for more than a century [12,13]. However, despite tremendous efforts, especially in the past forty years, rechargeable batteries are still far from being at their optimal performance [14,15]. In comparison to theoretical and estimated values, both present energy density and cost still offer plenty of room for improvements based on ongoing research and development [16,17]. The latter will be outlined with respect to ambient-temperature metal-air (technically described as “metal-oxygen”) batteries (MABs) in the following review.

Specifying the previous remark, throughout this review, “metal-air battery” is employed as a general term, even though the oxygen in question might not have been extracted from ambient air, due to the accompanying (challenging) reaction conditions. However, in order to still account for this essential difference, i.e., whether pure (O_2_-) or ambient oxygen (air-) was applied for the individual experiment, the differentiation between these two cases is maintained in terms of the individual abbreviation, i.e., Me-Air- or Me-O_2_-battery (Me denoting the individual metal of the system in question). Furthermore, the terms ‘*anode*’ and ‘*cathode*’ are set by the function of the individual electrode during the discharge of a full metal-air cell, following an imperfect but accepted convention in battery research [18].

### 1.1. Motivation for Metal-Air Batteries

Facing increasing user requirements regarding specific energy and power density as well as battery cost, environmental friendliness and safety, especially over the past four decades, battery research has become a much-noticed scientific field [19,20,21]. Since the 1980s, scientists created many novel battery concepts such as Li-ion batteries, which are state-of-the-art for mobile applications today and have become increasingly important for stationary applications as well [22,23,24]. However, although present Li-ion batteries provide up to 1000 Wh/kg on the material level [25,26,27] and have been projected to deliver up to 350–450 Wh/kg on cell level [28] at potential costs as low as $160/kWh by 2025 [16,28], Li-ion batteries still do not satisfy industrial needs completely, which is mostly due to the cost and high molar mass of the Li-ion host materials [29]. In 2010, the U.S. Department of Energy (DoE) estimated the cost requirements for batteries applied to grid scale energy storage facilities to be about $100/kWh to be competitive [30]. Moreover, the driving range of battery electric vehicles should be at least 350 km (215 miles) in order to be considered by a large number of costumers [31]. However, the latter will not be accomplished by every manufacturer very soon [28,32]. Therefore, fundamental research should also engage alternative battery concepts, aiming at extended electrochemical storage capabilities for large amounts of excess renewable energy and increased operating time/range for mobile electric devices [33,34,35].

**Figure 1:**
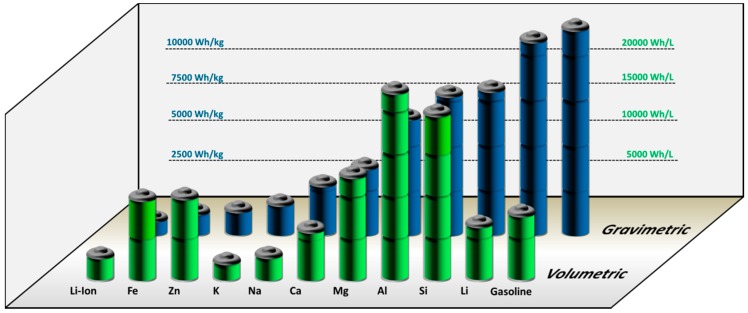
Theoretical energy content of various elements used for metal-air batteries in comparison to projected Li-ion battery cathode materials and gasoline. (Based on the reduced material, i.e., excluding oxygen uptake; Si is considered as a metal, since heavily doped Si is applied to Si-air batteries comparable to any other metal for metal-air batteries.).

Among all different types of potential next-generation (beyond Li-ion) batteries, room temperature metal-air batteries (MABs) with liquid electrolyte have attracted considerable scientific attention, owing to the following battery properties. First, MABs may potentially employ pure metallic anode materials, which feature excellent specific energies as well as excellent energy densities. Second, in comparison to conventional cells, MABs typically possess an open cell structure, which enables oxygen supply from ambient air and prevents the diminution of the overall battery energy content by electrode balancing. In MABs, the cathode reactant, oxygen, is (ideally) drawn from the outside and is not stored inside the cell, which eliminates the necessity of a bulky cathode material [36,37,38,39]. Illustrating the two advantages, Figure 1 depicts the specific energy of several solid MAB anode-materials calculated based on Faraday’s law in comparison to the specific energy of well-performing Li-ion battery cathode materials and gasoline:

W_Me/MeOx_ = n · F · M_Me/MeOx_^−1^ · U_cell_. (1)

W denotes the specific energy of the material. n denotes the number of transferred electrons. M_Me/MeOx_ denotes the molar mass of the material. U_cell_ denotes the standard full-cell voltage.

Applying Equation (1), the specific energies of Fe, Zn, K, Na, Ca, Mg, Al, Si and Li range from 1229 to 11430 Wh/kg (excluding oxygen uptake), which is summarized in Table 1 along with the open-circuit voltages of the corresponding MABs. Comparing the energy content of the reduced materials, the blue columns in Figure 1 highlight that the weight-specific values of all nine considered elements exceed the specific energy of present and projected Li-ion battery cathode materials, although losses due to electrode balancing in a Li-ion full-cell are not even included yet. Moreover, in Figure 1 it can be seen that the specific energy of Li is almost comparable to the specific energy of gasoline, which is as high as 11600 Wh/kg compared to 11430 Wh/kg for Li, making Li-O_2_ batteries particularly appealing for practical applications [40,41]. However, when taking the mass densities (*ρ_Me_*) of the individual elements into account, their distinct order according to the specific energies changes significantly, in favor of other elements than Li. Caused by its comparatively low mass density, the potential energy density of Li is relatively small compared to other elements. In fact, Fe, Zn, Mg, Al, and Si exhibit theoretical energy densities in the range from 9653 to 21837 Wh/L, which are (much) higher than those for Li and gasoline (6104 Wh/L and 8677 Wh/L). Accordingly, from the battery volume-perspective, the former five elements appear far more interesting than Li, if at least a good fraction of the theoretical energies is achieved on full-cell level.

Beyond the changing perception of individual elements based on energy density rather than specific energy, pros and cons regarding the energy content of individual metals change again if the oxidized instead of the reduced material is considered for the evaluation (Table 1). In case of the latter, Li metal particularly falls behind compared to the outstanding values in the reduced state due to the enormous relative weight gain during Li oxidation. Owing to the uptake of at least one oxygen atom per Li atom, the specific energy of Li drops by more than 50%, while other elements do not experience such a drastic change. In case of Zn and Fe for example, the given values for the specific energies decrease by only 20% and 38%, respectively, given the higher relative mass of the bare element compared to oxygen. Accordingly, the application of other metals rather than Li becomes increasingly interesting, if the specific energies in the reduced state are not the only parameter for the assessment of different systems.

A second aspect that should be within the scope of MAB-research is given by the rechargeability of the individual system. In fact, as implicitly included in Table 1, electrochemical rechargeability has not been shown for every metal yet. Until today, extensive electrochemical rechargeability has been shown for Fe-, Zn- and Li-electrodes, while rechargeability for the other systems appears to be far more challenging. However, limited rechargeability according to present knowledge is not the final argument against the investigation of a particular system. The development of primary Si-air batteries for example is driven by the second highest energy density of 19748 Wh/L as well as descent resource-efficiency for Si.

### 1.2. Resource-Efficiency

In context with climate change and the pursuit of renewable energy sources, it is self-evident to consider the sustainability of an energy system including storage and distribution as a whole, instead of the energy harvesting technology alone. Otherwise, the evaluation in comparison to currently almost flexibly applied fossil fuels would be incomplete and misleading. Conventional technologies do not require storage facilities to a great extent, but intermittent renewable energy sources do. Hence, resource-efficiency and crustal abundance must clearly be within the scope of battery research, aiming at an eco-friendly and sustainable energy system. A truly sustainable energy system must be safe from material shortages and must not rely on aggressive extraction methods for the materials of its key components. Both are not the case for Li-ion batteries. In fact, state-of-the-art Li-ion batteries face a potential lack of Li- and Co-supply in the future, while Co is also considered to be extracted under questionable circumstances [29,56,57].

**Figure 2:**
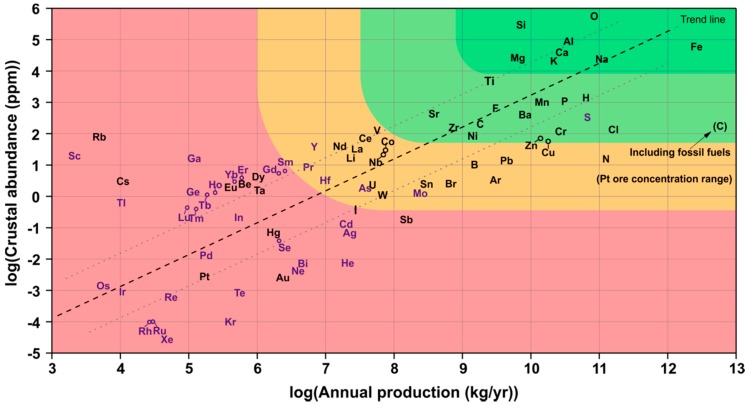
Estimated crustal abundance for all natural elements on Earth plotted as a function of annual production. Elements which are main economic products of their respective ores are shown in black font, while purple font is used for the elements, which are mostly byproducts of other elements. Green color indicates abundant, readily available elements. Light green and orange indicate less abundant elements. Red color indicates scarce elements. (Reprinted and adapted with permission from RSC Adv., 2, 7933–7947, ©2012, The Royal Society of Chemistry.) [58].

Considering resource availability with respect to large-scale industrial applications, crustal abundance of the required elements is a first indicator for the potential success of an emerging technology. Figure 2 shows the estimated crustal abundance for all natural elements on earth plotted as a function of present annual production [58]. Indicated by the different colors, from Figure 2 it can be derived that there are only very few elements that are readily available in a large amount. These elements are namely: Si, Al, Fe, Ca, Na, K, and Mg, which exhibit a crustal abundance above 10^4^ ppm while also being extracted at a rate of more than one million metric tons per year. In this sense, particularly the production of Fe is extremely high and exceeds the production of almost every other element with an annual amount of 1.3 billion metric tons. Only the production of carbon due to the exploitation of fossil fuels is higher [59]. In contrast to that, Zn and Li are far less abundant, although their production is still not as critical as the production of some rare-earth elements like Gd or Dy (magnet production), of course. Featuring an abundance of about 10^2^ ppm and 10^1^ ppm, respectively, Zn and Li are at least 100 times less abundant than Al, Fe and Si, which makes the latter three elements highly interesting for practical applications in resource-efficient batteries. In addition, the application of Zn for the use in batteries competes with several other applications such as the corrosion protection of steel, which might become an issue due to potentially increasing Zn prices in the future [60,61]. However, providing a long-lasting rechargeable Zn-air battery would, of course, be a major achievement in view of countless sold, but barely recycled Zn-based batteries worldwide [62]. In this regard, it has recently been explained by Clark et al. that Zn-air batteries are on their way to better performances as predicted by continuum modeling, which has just been proven by Wang et al. again [63,64]. Furthermore, the same arguments apply to the research on Li-O_2_ batteries. High-performance rechargeable Li-O_2_- and, finally, maybe even electrochemically rechargeable Li-air batteries would most probably replace Li-ion batteries in the long-run, increasing the impact but, maybe, aggravating the availability of Li in the future. Due to the increasing need of Li for Li-ion batteries, the Li price rose by several hundred percent over the past two decades already [65] and is expected to rise even further as soon as the share of annually sold electric vehicles will climb far above 1% (2017) [66].

**Figure 3:**
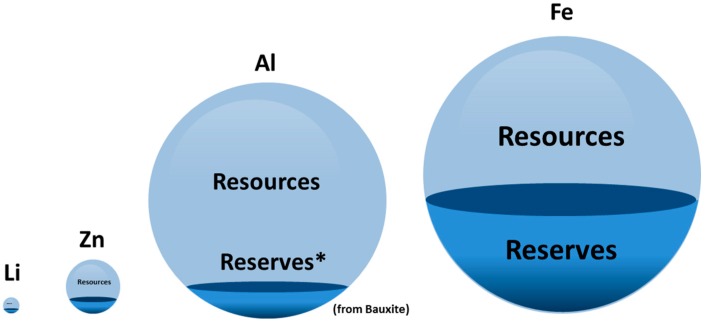
Relative availability of Li, Zn, Al, and Fe based on the estimated amount of “resources” and “reserves” in the Earth’s crust represented by the relative volume of the spheres (U.S. Geological Survey 2017) [59]. (*No clear calculations available; reserves for Al are considered to supply the worlds demand constantly—No definite estimations available for Si).

Beyond crustal abundance, a second major quantity that should be considered discussing the resource-efficiency of certain battery electrode materials is given by the estimated amount of “resources” and “reserves”. Following the definition by the U.S. Department of the Interior, “resources” consider the known amount of material, which is feasible or potentially feasible to be extracted by present extraction techniques. In contrast to that, “reserves” consider the working inventory of mining companies only and might, therefore, be far lower than the actual resources, if only a few known deposits were explored [67]. Considering “resources” and “reserves” for Fe, Si, Al, Zn, and Li, from annually updated estimations it can unambiguously be established that Fe, Si, and Al are set to supply the worlds demand for many decades or even centuries [59]. According to the U.S. Geological Survey 2017, the world reserves for Fe amount up to at least 82 billion metric tons (230 billion metric tons of resources), while the annual production in 2016 was only 1.3 billion metric tons with the main demand resulting from the construction sector. In contrast to that, the world reserves of Li were only about 14 million metric tons (40 million metric tons of resources), while the annual production was 35 thousand tons, with the main demand originating from the battery industry by far already (Until 2017: 2 million sold electric vehicles worldwide out of about 1 billion sold vehicles since the year 2000 [66,68]). Displaying the discrepancy between Fe, Al, Zn, and Li in terms of “resources” and “reserves” in a 3D plot shown in Figure 3 (Si: definite estimations are not available), it becomes clear that a broad transformation of the global electricity supply might eventually require more abundant materials than Li. In fact, Li might not cover the extensive and rapidly growing demand for energy storage materials as resource-efficient, highly available elements such as Fe, Si, and Al could do. Furthermore, at least for Al and Fe, a recycling industry has already been established, while Li-ion battery recycling appears to be complicated and is under urgent development in order to cope with large numbers of exhausted Li-ion batteries in the future [69].

### 1.3. Challenges for Metal-Air Batteries

For MABs, from a theoretical point of view, the energy content of the cell is exclusively determined by the amount of available anode material, since the other electrode reactant, oxygen, is, ideally, drawn from ambient atmosphere [70]. Furthermore, it is assumed that the air electrode is infinitely thin, which is highly beneficial for the theoretical energy density of the battery, since the battery volume is not reduced by a second electrode material with considerable dimensions. However, the application of oxygen and particularly the application of oxygen from ambient atmosphere requires an at least partially open battery casing, which inflicts the risk for the electrolyte of drying or leaking out [71]. Moreover, the intrusion of CO_2_ and moisture may cause detrimental side reactions like the carbonation of alkaline electrolytes or the decomposition of the anode material due to reactions with water, which has to be resolved in order to prevent MABs from premature failure [36,37]. In this regard, especially the customized design of the air electrode by the application of appropriate membranes has proven to be effective, but still offers room for improvements [37]. Moreover, further challenges for MABs remain in the following issues, which will briefly be discussed depending on the applied type of electrolyte, i.e., aqueous or non-aqueous electrolyte.

In case of *aqueous electrolytes*, which mainly apply to Fe-, Zn-, Al-, and Si-air batteries, major challenges for MAB cathodes persist in the implementation of cheap, non-noble, but, ideally, bifunctional, high-performance catalysts for the oxygen reduction and the oxygen evolution reaction (ORR/OER). In fact, the ORR is a sluggish reaction, which reduces the availability of appropriate non-noble catalysts already and decreases the choice of material even further as soon as high-performance OER is required for the same material [72,73]. However, the application of bifunctional catalysts avoids the risk of rapid catalyst degradation and, with this, the need for two separate air electrodes for charge and discharge, respectively. Catalyst degradation during repeated electrochemical cycling of a MAB particularly occurs if two separate catalysts are chosen for the individual functionality of ORR and OER on the same electrode. In this case, a fairly wide potential range applies to both materials under which the individual ones might not be stable and, eventually, necessitate the unfavorable, since far more complex application of two separate air electrodes (three-electrode configuration) [44,74]. Thus, dedicated catalyst research with respect to bifunctional materials is of major interest for MABs in order to reduce the complexity of the battery, and actually the most frequently reported scenario for aqueous MAB research in literature [75,76,77].

**Figure 4:**
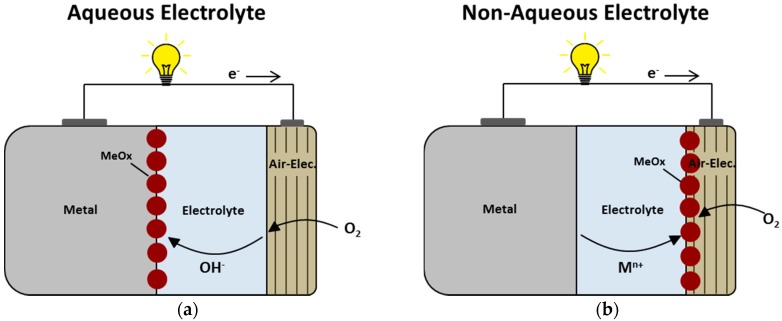
Schematic representation of the reaction zone in (**a**) aqueous and (**b**) non-aqueous metal-air batteries.

For aqueous MAB-anodes, three major issues are seen in the non-uniform dissolution and reprecipitation of the anode material as a metal oxide or -hydroxide on the metal electrode surface during repeated electrochemical cycling (Figure 4a), the inherent tendency of most metals towards spontaneous corrosion, and the hydrogen evolution due to water splitting upon recharge of the battery. Among the three, the first issue may result in dendrite formation, which is particularly obvious for Zn-air batteries and occasionally leads to internal short circuits in the battery, if the applied current density during the recharge is too high [78]. The second issue may result in irreversible discharge capacity losses due to the wasteful dissolution of the anode material in the electrolyte. Particularly in concentrated alkaline electrolytes, which are preferred for most aqueous MABs due to their excellent ionic conductivity, most anode materials show severe corrosion unless a passivating layer forms on the electrode surface and prevents the metal from gradual dissolution on standby [71]. Third, the extensive hydrogen evolution due to water decomposition should be avoided in order to prevent the battery from drying out and to increase the coulombic efficiency by the elimination of a parasitic side reaction during the recharge of the battery [70].

In case of *non-aqueous electrolytes*, which particularly apply to Li-, Na-, K-, Mg-, Ca- and Si-air- and (-O_2_) batteries, (respectively), one of the most important challenges in comparison to aqueous MABs remains in the quite different reaction zone for the discharge products. In non-aqueous MABs the discharge products typically accumulate on the air electrode (Figure 4b), which requires the air electrode to provide large storage capacity as well as decent oxygen permeability to be effective for long-lasting battery discharge. Otherwise, the discharge of the cell will stop prematurely, as the battery dies from oxygen starvation as soon as the discharge products clog the pores of the air cathode and prevent the influx of additional oxygen into the battery [36,38,79]. Moreover, in non-aqueous MABs, also a suitable way to lower the cathode-related overpotential during the recharge has to be identified, in order to maintain the performance and achieve long-term cycling stability [39]. The latter may be approached by the determined design of the oxygen catalyst, which is, however, a challenging field of its own, since the exact pathway of the cathode reaction depends on the non-aqueous electrolyte and might not be clear in every detail [80].

Furthermore, depending on the individual anode material, a suitable electrolyte that is stable against decomposition by all of the occurring charge-/discharge (by-)products has to be identified, which is a demanding task due to the marked reactivity of the individual chemicals [81,82,83]. For Li-O_2_ batteries, for example, it has recently been observed by Wandt et al. that the formation of singlet oxygen as a byproduct during the battery recharge might be the long overlooked link in the understanding of the electrolyte degradation [84]. Another possible degradation mechanism for non-aqueous electrolytes can be corrosive reactions with the applied electrode material, which result into capacity losses of the battery and a possible growth of a passivation layer on the metal surface [85,86]. Moreover, for secondary MABs, the non-uniform re-deposition of the metal upon recharge may lead to dendrite formation, which, eventually, causes short-circuits and shedding of the electrode. In case of highly reactive metals as anode materials, for safety issues, it is important to prevent water and oxygen access to the metal itself while forming stable SEI in order to inhibit electrolyte decomposition on the metal surface [87,88,89,90].

### 1.4. Electrochemical Performance of Metal-Air Batteries

**Figure 5:**
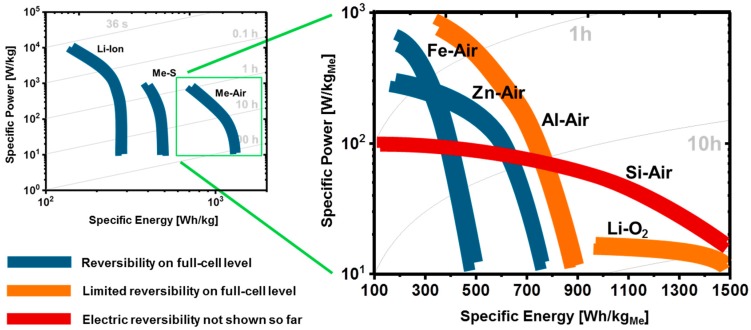
Ragone plots for Li-ion-, metal-sulfur- (Me-S-), and ambient temperature metal-air batteries (MABs). Left panel: Ragone plot for the general comparison of present Li-ion-, Me-S- and MABs. Right panel: Ragone plot as a comparison for the potential performance of different MABs, roughly estimated based on the individually best results reported for MAB anodes and MAB cathodes in literature (cf. Section 1.4) [41,52,54,94,95,96,97,98,99].

Considering the performance of MABs based on half-cell- and non-optimized full-cell experiments (since there are very few true full-cell results yet), it can already be established that Fe-air-, Zn-air-, Al-air-/Al-O_2_-, Si-air-, Li-air-/Li-O_2_-batteries may provide excellent specific energy as well as medium specific power on full-cell level in the future. Illustrating the potential performance of MABs in comparison to Li-ion- and metal-sulfur- (e.g., Li-S-) batteries, in the left panel of Figure 5 it is shown that state-of-the-art Li-ion batteries provide specific energies in a range from 200 to 300 Wh/kg and a maximum specific power of up to 10 kW/kg [25]. In contrast to that, sophisticated metal-sulfur batteries currently deliver specific energies of up to 500 Wh/kg and specific power of up to 1 kW/kg [91,92]. Furthermore, estimated values for the performance of potential MABs range from 500 to 1500 Wh/kg_Me_ in terms of specific energy and from 0.1 to 1 kW/kg_Me_ in terms of specific power [41,52,93,94].

However, while a comparison of Li-ion-, metal-sulfur- and MABs in general is comparatively straight-forward, a fair comparison between different types of MABs is rather complicated, owing to the individual limitations of the different systems and a multitude of experimental parameters. Due to these, first of all, the energy and power of MABs are either given normalized to the electrode area or the active weight. Furthermore, depending on whether the metal- or the air electrode is the limiting instance (cf. Figure 4), the battery performance is either given in terms of anode- or cathode-related values, which need to be converted into each other to be comparable. As a method for this conversion, in this review the actual limitation is, first, expressed in terms of its experimental value (i.e., given in Wh), which is than matched by an equal value for the considered limitation assuming plane, non-porous material, 100% conversion efficiency and no additional material to be required for the reaction (e.g., Wh/cm^2^_Carbon_ → Wh/kg_Me_).

Applying the procedure described in the previous paragraph, a comparable but fairly rough estimation for the performance of different MABs in terms of a Ragone plot can be derived. However, unlike the specifications for Li-ion-, Me-S- and MABs in general (left panel, Figure 5), the estimations in the right panel of Figure 5 must not be understood as readily available, but potential future performances of Fe-air-, Zn-air-, Al-air-, Si-air-, and Li-O_2_ batteries. Almost none of the displayed values, except for the performance of Si-air batteries, can be found for a single cell reported in literature. The estimated performances rather provide an overview how the general capabilities of the different systems compare to each other, based on the (virtual) combination of the individually best-performing anodes and cathodes reported in literature. Further information about the actually reported performance as well as potential limitations can only be considered separately for each system, which is provided by the summary of recently reported results in Table 2. (As a special feature, Table 2 particularly provides the conditions under which the individual results were obtained. From the comparison of the conditions it can immediately be derived, that the individual investigations differ significantly in terms of experimental scope, enabled by the multitude of possible experimental parameters. E.g., for the determination of the maximum performance, typically, only single, deep-discharge step is applied to the investigated system, while its reversibility is tested for a minimum depth-of-discharge (DoD) in every cycle.).

**Figure 6:**
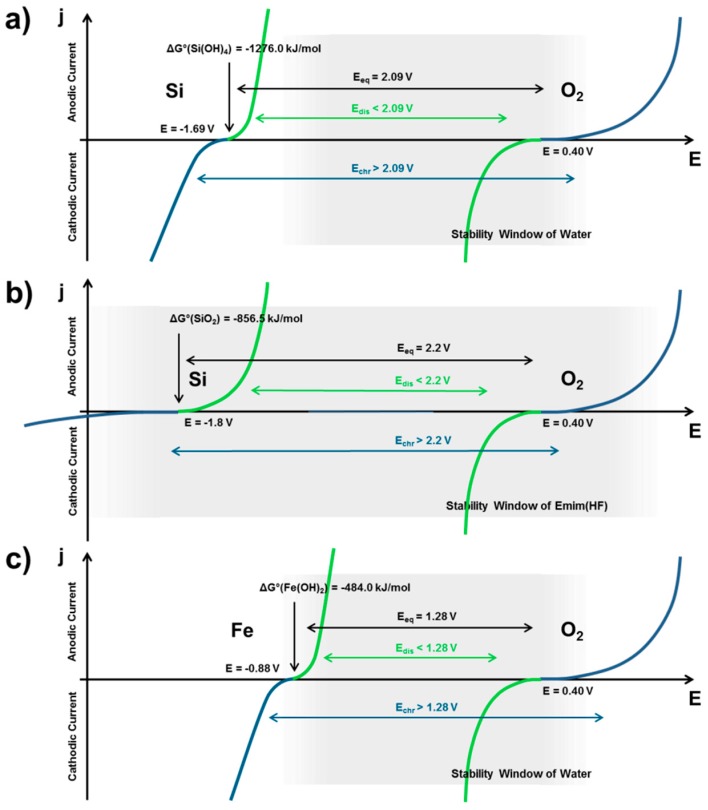
Polarization curves and cell chemistry of (**a**) aqueous Si-air-, (**b**) non-aqueous Si-air- (e.g., EMIm(HF)_2.3_F) and (**c**) aqueous Fe-air batteries. Grey areas indicate the stability window of the corresponding electrolyte, i.e., water or room temperature ionic liquid (RTIL), respectively. Green color indicates discharge conditions, blue color indicates charging conditions, black font indicates equilibrium conditions. (Adapted from [36]).

From the comparison of the estimated performances of potential MAB full-cells in Figure 6 it can be concluded that Fe- and Zn-air batteries provide the highest reversible specific power among the five considered MAB systems yet. The latter makes both of them an excellent, even complementary choice for practical application in the future [93,94,95,96,97,100]. While Zn-air batteries typically provide higher specific energies upon single discharge, Fe-air cells exhibit superior reversibility even at repeated complete (100% DoD) discharge of the battery (cf. Table 2). However, particularly in terms of specific energies, Fe- and Zn-air batteries are still not the last word in MAB research. Already today, experimental Al-air-, Si-air-, and Li-O_2_ batteries exceed the performance of Fe-air and Zn-air batteries in terms of specific energy, but still fall behind, owing to their limited rechargeability as well as potential scale-up issues. Present Al-air- and Li-O_2_ cells exhibit specific discharge capabilities of up to 1500 Wh/kg_Me_ and more, but are only rechargeable for a few tens of cycles, yet [41,55,98,105]. Furthermore, in case of Si-air batteries, electrochemical rechargeability has not been shown yet, which is, however, no reason to discontinue the research on this type of battery [50,52]. Considering primary applications, silicon-based batteries from scrap material could be a decent option to replace primary Zn-air batteries in the future, given the excellently flat and long-lasting discharge characteristics of silicon in comparison to zinc. Furthermore, similar to Ca-O_2_ batteries, it does not appear ultimately impossible to tackle the missing rechargeability of Si-air batteries [106,107,108], which is effected by thermodynamics and exemplarily explained with the help of polarization curves in comparison to rechargeable Fe-air batteries in Figure 6.

Based on thermodynamic considerations, the currently missing reversibility of Si-air batteries compared to other metal-air systems arises from two major issues. First, the exceptional stability of the discharge products, which are either Si(OH_4_) (or silicates) in alkaline electrolyte (ΔG(Si(OH_4_)) = −1276 kJ/mol) or SiO_2_ in room temperature ionic liquids (RTILs) like EMIm(HF)_2.3_F (ΔG(SiO_2_) = −856.5 kJ/mol) and, second, the extremely sluggish kinetics of its reduction indicated by a comparatively flat polarization curve (cf. Figure 6a,b) [52,53]. Due to the exceptional stability of the oxide materials, the equilibrium electrode potential of silicon is fairly low, which is critical for the stability of the electrolyte. In contrast to iron, the electrode potential of silicon in alkaline electrolyte is far out of the stability window of water (grey area in Figure 6a), which results in the evolution of hydrogen due to water decomposition instead of the reduction of the silicon electrode in alkaline electrolyte [52]. In case of iron, the equilibrium electrode potential typically lies at the edge of the stability of water (Figure 6c; ΔG(Fe(OH)_2_) = −484 kJ/mol), which is also critical but still acceptable if the hydrogen evolution is effectively addressed by electrode or electrolyte additives like Bi_2_S_3_ or Na_2_S (cf. Section 3.7) [109]. Furthermore, due to the sluggish kinetics of the SiO_2_ reduction in EMIm(HF)_2.3_F, the overpotential of the silicon electrode during the recharge increases rapidly. While the equilibrium potential of silicon in EMIm(HF)_2.3_F lies well within the stability window of the electrolyte, the RTIL might easily decompose upon recharge, if the reduction potential is shifted to critically low values. The electrochemical deposition of Si in other RTILs have already been reported [106,107,108], which might enable the rechargeability of Si also on a battery level in near future.

Beyond the thermodynamic stability and the sluggish reduction, the reversibility of silicon is also hindered by the spontaneous corrosion of the anode material. The corrosion of silicon is particularly obvious in alkaline electrolyte and far more extensive compared to other systems like alkaline Fe-air batteries. This is, however, no exclusion criterion for the implementation of silicon-air batteries [110]. In fact, the application of corrosion inhibitors offers a promising path towards much higher (cycling) efficiencies [38,111,112]. Moreover, the development of highly reactive, electrochemically resistant electrolytes as well as the development of metal electrodes with low overpotentials could, someday, result in an improved battery performance after all.

The following two sections of this review will focus on the application of silicon and iron as resource-efficient anode materials for metal-air batteries. The latter is particularly motivated by the excellent availability of silicon and iron in the Earth’s crust, which will guarantee almost infinite supply of both environmentally friendly electrode materials. Furthermore, it is clear that silicon-air batteries provide an outstanding energy density (at least) for primary applications, while iron-air batteries can be considered as a rechargeable metal-air battery system with potentially high power densities. However, while there are several excellent reviews about other metal-air battery systems such as zinc-air- [44,113,114,115] and lithium-air [36,40,81,116,117], as well as for metal-air batteries in general [37,38,79,118], very few has been written about silicon- and iron-air batteries. To the best of the author’s knowledge, only the review by Gelman et al. is concerned with (non-aqueous) silicon-air batteries [50], while there is also only one recent minireview about iron-air batteries written by McKerracher et al. [119]. Accordingly, in the present review, we will focus on aqueous silicon- and iron-air batteries.

## 2. Silicon-Air Batteries

### 2.1. Overview

Besides being the second most abundant element in the Earth’s crust, the particular property of Si as a semiconductor makes it a unique candidate for electronics; but despite possessing a good potential, until 2009 no attempts have been reported to use silicon as an anode material in batteries. Thereby, Si has been one of the most investigated material in the field of semiconductor technology over the last five decades [120,121,122]. The first attempt to utilize highly doped Si (dopant concentrations ~ 10^19^ 1/cm^3^)—doping is essential for electronic conductivity—as an active electrode in a metal-air battery concept, however, was introduced by Ein-Eli [123]. On the basis of theoretical considerations, the Si–O_2_ redox-couple is very encouraging for battery applications in terms of specific energy related to Si mass—when Si is oxidized to Si^4+^. The first Si-air cell was constructed by employing a non-aqueous electrolyte—1-ethyl-3-methyl-imidazoliumoligofluorohydrogenate (EMIm(HF)_2.3_F). Such a novel room-temperature ionic liquid (RTIL) exhibits unique properties such as wide electrochemical window, high ionic conductivity (100 mS/cm), low viscosity, and chemical stability in air [124,125,126]. More recent investigations, on the other hand, focused on conventional aqueous alkaline electrolyte—potassium hydroxide (KOH) [52,127,128]. Due to its superior ionic conductivities, especially between 5M–8M KOH (>600 mS/cm) [129], KOH solutions have extensively been used as electrolytes for Zn-air-, Al-air-, and Fe-air batteries [36,38,44,54,119]. Considering the already existing semiconductor-related studies, where NaOH or KOH solutions have widely been employed for etching or anodization of Si [121,130,131,132,133,134,135,136,137,138], such cost effective alkaline solutions are potentially promising also for Si-air batteries. Additionally, some attempts have been succeeded to establish solid state (or polymeric gel based) electrolytes in Si-air batteries with reasonable discharge performance [139,140,141]. An overall summary of the possible Si-air systems is provided in Table 3. This review, however, focuses only on the liquid based (aqueous and non-aqueous) electrolytes.

In general, both aqueous and non-aqueous electrolytes provide the same fundamental mechanisms on Si and the air electrode, i.e., Si is oxidized at the anode and O_2_ is reduced at the cathode. The exact reactions, however, show substantial differences in each solution. Hence, both systems will be considered individually within the next sections. 

### 2.2. Aqueous Alkaline Si-air Cells

**Figure 7:**
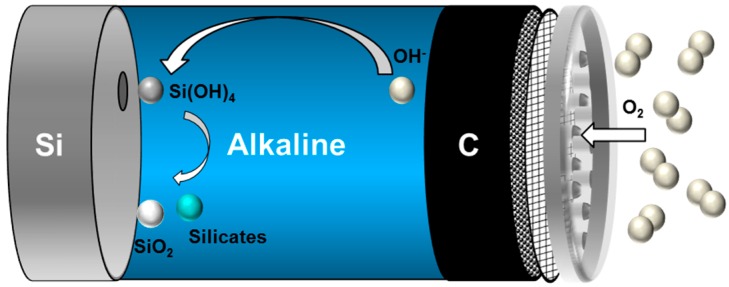
Schematic representation of the general processes upon the discharge of Si-air batteries with alkaline electrolytes.

The basic structure of the primary Si-air cell is illustrated schematically in Figure 7. The cell is comprised of three main parts: A silicon wafer as anode, alkaline solution as electrolyte, and a carbon based air electrode as cathode. During the discharge of the cell, Si is oxidized at the anode producing four electrons and silicic acid (Si(OH)_4_) as reaction products (Equation (2)). Depending on the pH level of the alkaline electrolyte, silicic acid is then ionized to either SiO(OH)_3_^−^ (pKa = 9.5) or SiO_2_(OH)_2_^2−^ (pKa = 12.5) [145,146]. In the presence of weak alkaline solutions (pH < 9) or neutral solutions, silicic acid would be converted into SiO_2_. Concomitant to the Si oxidation, diffused oxygen from the air is reduced by the catalysts at the cathode (Equation (4)). Subsequently, generated hydroxide ions migrate to the silicon anode and maintain the oxidation reaction. The electrochemical reactions upon discharge of a Si-air cell are described as [121,127,132]:

Anodic         Si + 4OH^−^ ⇌ Si(OH)_4_ + 4e^−^              E^0^ = −1.69 V (2)

Formation of silicate:  Si(OH)_4_ + 2OH^−^ ⇌ SiO_2_(OH)_2_^2−^ + 2OH^−^               (3)

Cathodic:         O_2_ + 2H_2_O + 4e^−^ ⇌ 4OH^−^              E^0^ = 0.40 V (4)

Corrosion:       Si + 2OH^−^ + 2H_2_O ⇌ SiO_2_(OH)_2_^2−^ + 2H_2_               (5)

From the thermodynamical considerations, the half-cell potentials of anode and cathode are −1.69 V and 0.40 V vs. SHE, respectively. Thereby, the alkaline Si-air cell theoretically has a standard cell voltage of 2.09 V (considering a 4 electron process). In practice, however, the cell can only provide 1.50 V open circuit voltage (OCV) and be operated at voltages around 1.20 V under relatively low discharge current densities of 50 µA/cm^2^ [52,127].

There exist several challenges limiting utilization of Si-air batteries more efficiently, such as reversibility, corrosion and electrolyte management. Reversibility of the system has not been shown yet due to (i) stability of SiO_2_ if present as an end product, (ii) complexity and stability of silicate chemistry in concentrated alkaline solutions. Moreover, during the operation of Si-air cell, Si undergoes corrosion (Equation (5)) simultaneously with the oxidation reaction. As Si is rather an active element, especially in alkaline media, corrosion reaction starts already at the OCV spontaneously and continues in parallel to discharge. According to Equation (5), corrosion of Si involves an attack by water and hydroxide ions producing soluble silicates and hydrogen gas. Both reaction products could be detrimental to the battery performance due to (i) formation of viscous solutions when silicates are in high concentrations, (ii) increase on the cell pressure leading to possible electrolyte leakage, and (iii) consumption of the active material. Regarding the last point, the kinetics of the corrosion reaction are remarkably fast which result in only few percent of mass conversion efficiencies (~3%) in aqueous alkaline Si-air cells [52]. It is, therefore, of high importance to investigate corrosion mechanisms of Si in order to understand the possible influences on the battery shelf-life, discharge performance, and efficiency.

#### 2.2.1. Thermodynamics of Si in Aqueous Electrolyte

**Figure 8:**
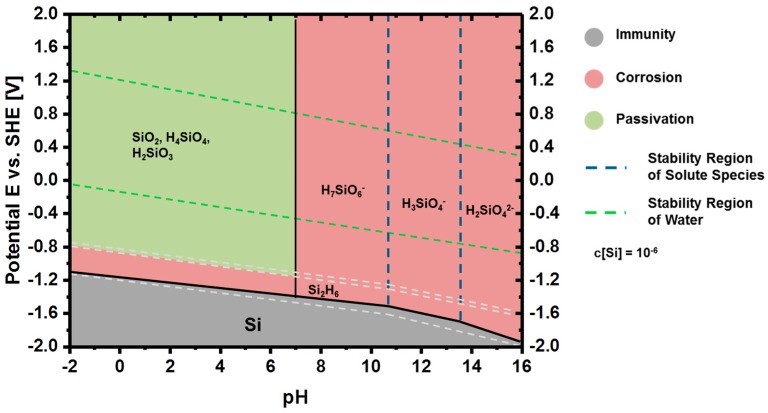
Pourbaix diagram of Si in aqueous solutions at 25 °C (after Pourbaix and Nikolaychuk [147,148]).

The thermodynamic stability of silicon in aqueous solutions and water is represented graphically by potential-pH diagrams (Pourbaix diagrams) [147,148]. According to Figure 8, silicon is not stable in water and aqueous solutions, since its immunity region remains far below the stability region of water. Thereby, in such solutions, silicon is prone to instant oxidation, while forming silica, silicates, or other products depending on the solution pH.

Pourbaix diagrams are solely based on the thermodynamic properties of the elements; there is no experimental evidence for some of the species shown in Figure 8 [148]. Nonetheless, such diagrams identify the individual regions where the element of interest shows immunity, passivity, or corrosion behavior. In general, when the stable specie in a region is a dissolved ion, i.e., H_7_SiO_6_^−^, H_3_SiO_4_^−^, or H_2_SiO_4_^2−^, this region is considered as “corrosion region”. On the contrary, when the stable specie is in the form of a solid oxide or solid hydroxide, i.e., SiO_2_, the region is labeled as “passive region”. If the element of interest remains unreacted with the solution, this region is an “immune region”. Accordingly, these regions for Si are illustrated in Figure 8. It is clear that Si is thermodynamically not stable in alkaline solution (i.e., pH > 13); hence, it reacts with the solution spontaneously forming silicates as SiO_2_(OH)_2_^2−^ and H_2_ gas according to Equation (5). Any insight into reaction kinetics, however, cannot be provided by Pourbaix diagrams, since they are solely based on the thermodynamic properties of the elements under equilibrium conditions.

#### 2.2.2. Corrosion Behavior of Si in Aqueous Alkaline Electrolyte

The kinetics of the Si reaction mechanisms in aqueous alkaline media, mainly as dissolution (etching) rate and passivation (anodic oxide formation), have already been subject to many investigations in the fields of semiconductor and micromachining [120,121,122,130,132,133,138]. The studies were generally based on etching of crystalline Si in KOH or NaOH at open circuit potential as well as under anodic potentials to obtain desired specifications (i.e., surface characteristics) of Si. The influence of Si crystal orientations on etching kinetics revealed practical importance of such alkaline solutions as anisotropic etchants for many systems [121,149,150]. In the view of a possible battery application by using Si as active anode, the dissolution or etching reactions would be accounted as parasitic processes; hence, “dissolution or etch rates” are referred as “corrosion rates” in the scope of this study.

**Figure 9:**
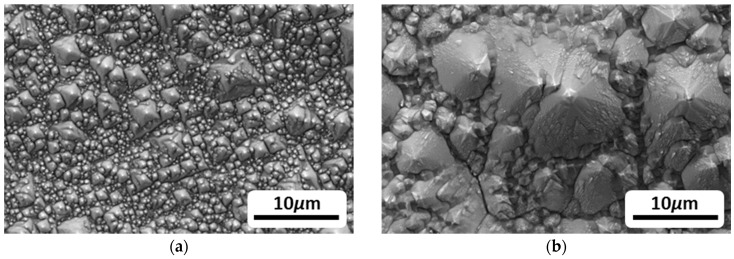
Typical pyramidal hillocks on As-doped <100> Si wafers upon immersion in different conventional aqueous alkaline electrolyte—potassium hydroxide (KOH) electrolytes for 24 h OCP at room temperature: **a**) 2M KOH, **b**) 5M KOH.

Generally, most of the investigations employed KOH solutions due to their high <100>/<111> etch ratios and nontoxic nature [120,150], although the major characteristics of the reaction processes that are responsible for Si etching in alkaline solutions are similar. Figure 9 illustrates the typical surface morphologies of Si after 24 h immersion in 2M and 5M KOH electrolytes. Due to the anisotropic etching of Si in such solutions, the major crystal orientations are selectively etched; therefore, there is a difference on the removal rates of surface Si atoms at different sites. As a result, surface morphologies such as pyramidal hillocks are formed on Si wafers as shown in Figure 9. The size of the pyramidal hillocks is dependent on the KOH concentrations due to the variations of the corrosion rates in differently concentrated KOH solutions [110].

The detailed mechanisms of the Si corrosion reactions are extremely challenging, which limits a deeper understanding of the processes; in particular, the nature of the reaction mechanisms. A differentiation between the chemical and the electrochemical mechanisms considers whether the charge carriers are involved in the reaction (electrochemical) or not (chemical). On the basis of independent etch rates on dopant type (n- and p-type Si) and dopant densities (up to 10^19^ 1/cm^3^), Glembocki et al. and Palik et al. argued that the etching mechanism of Si in alkaline solutions is in chemical nature [132,133,135,151]. Nonetheless, the supportive arguments in favor of electrochemical mechanism are (i) the etch rates are influenced by the applied potential, and (ii) the OCP and passivation peak potential (V_P_) are different for n- and p-type Si as well as for different dopant concentrations [133,152]. Seidel et al. discussed the Si dissolution mechanism by the help of energy band diagrams and energetically favorable surface states, which, consequently, lead to the proposal of an electrochemical mechanism [121]. According to Allongue et al., on the other hand, both chemical and electrochemical mechanisms co-exist and compete with each other during Si dissolution. Nevertheless, the chemical path of the reaction is more dominant, as the electrochemical etch rate is at least two orders of magnitude lower than the chemical etch rate [138].

**Figure 10:**
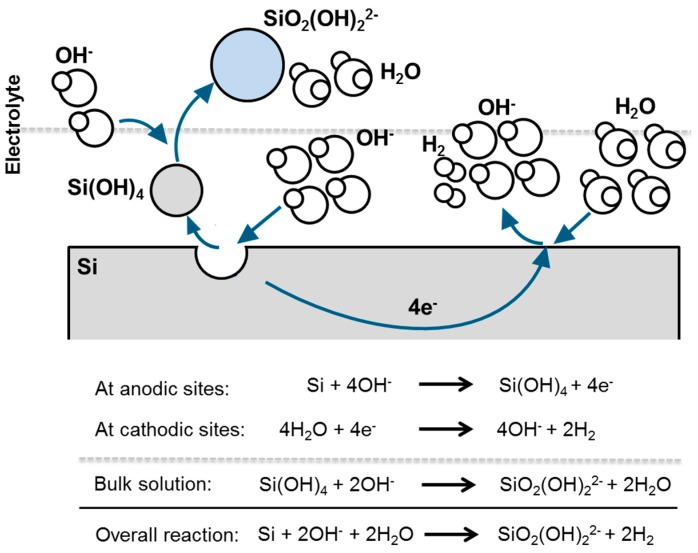
Schematic representation of the electrochemical corrosion reactions of Si (coupled) at open-circuit potential (OCP).

In general, most of the metals corrode via coupled electrochemical reactions. Figure 10 illustrates this behavior for a Si surface immersed in alkaline media. On the same silicon surface there is, on the one hand, a Si oxidation occurring at temporary anodic sites, on the other hand, concomitant water reduction takes place at temporary cathodic sites. During the oxidation reaction, Si dissolves and passes into solution as Si^4+^ (Si(OH)_4_). The produced electrons in this process are transported to cathodic sites where they are consumed by water reduction reaction. Overall, there is a material dissolution which is quantified by the anodic and cathodic reaction rates as well as by the electron flow between the different sites.

**Figure 11:**
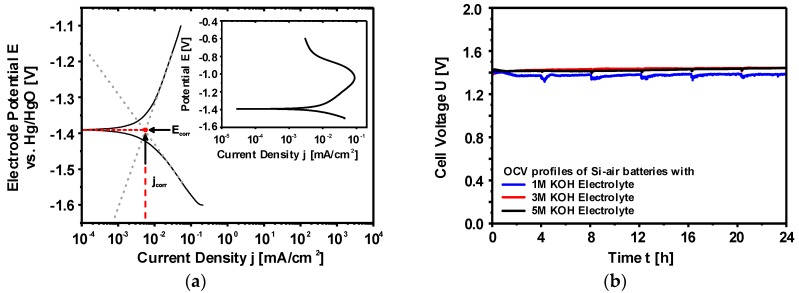
(**a**) Typical potentiodynamic polarization curve of Si in alkaline media in a small potential range where the Tafel slopes and corrosion current densities are calculated. A full scan is given as inset figure which depicts the typical passivation behavior. (**b**) The influence of the corrosion on the open circuit voltage of Si-air battery over 24 h.

One of the most common ways to measure the corrosion rate is the electrochemical polarization method which follows the mixed potential theory basing on the Butler-Volmer equation [153,154,155]. A typical potentiodynamic polarization curve of Si in alkaline media is depicted in Figure 11a. The potential scan starts from the cathodic potentials towards the anodic direction. At the plateau (corrosion potential or rest potential) shown in Figure 11a, the cathodic reaction rate is in equilibrium to the anodic reaction rate; hence, there is no net current flowing in the system. However, there is continuously a material dissolution. The rate of Si dissolution (corrosion) could be determined from the extrapolations of Tafel slopes that are obtained from the linear regimes of both anodic and cathodic curves. The intersection of slopes at the corrosion potential provides the corrosion current and accordingly the corrosion rate is calculated. Typical corrosion rates and current densities of highly As-doped <100> Si (n-type) in 5M KOH can be obtained around 6 nm/h and 5.3 µA/cm^2^ when electrochemical polarization method is applied [110]. These values might vary depending on the specifications of Si and alkaline solutions [138]. Additionally, the inset figure represents a wider potential scan in which Si shows a surface passivation phenomenon at higher anodic overpotentials. Under these conditions, the dissolution rate of Si(OH)_4_ into silicates is lower than its production rate; eventually, Si(OH)_4_ would be converted to SiO_2_ [121,127]. Consequently, as shown at potentials beyond −1.0 V vs. Hg/HgO (inset figure) the anodic current decreases when the Si surface is covered completely with the passive oxide layer. The formed SiO_2_ layer, however, would be dissolved revealing active Si surface upon the applied anodic potential is released and enough resting time is assured [138].

It is clear that polarization method considers only the electrochemical coupled reactions for the corrosion analysis. In case there are other reactions that are chemical in nature, polarization method may underestimate the corrosion parameters. If the corrosion products are non-adherent to surface, weight loss or etch depth measurements would be applicable to monitor more accurate corrosion behavior. In this regard, Palik et al. and Seidel et al. employed the etch depth method for the etching (corrosion) investigations of Si under various conditions in their studies [121,134,136,152,156]. For instance, the corrosion rate of n-type Si in 5M KOH was reported around 2400 nm/h by Palik et al. [156]. There is evidently a significant difference between the corrosion rates obtained from polarization method (6 nm/h) and the etch depth method (2400 nm/h). This difference was first emphasized by Allongue et al. who reported an equivalent dissolution current density of 799 µA/cm^2^ (from weight loss method) and an exchange current density of 3.7 µA/cm^2^ (from polarization method) [138]; hence possibly two different corrosion mechanisms were present. Note that all the corrosion investigations related to semiconductor field were almost exclusively conducted with low to medium doped Si wafers (dopant concentrations up to 10^16^ 1/cm^3^) since Si degenerates in case the dopant concentrations are too high (>10^20^ 1/cm^3^). In a recent study, on the other hand, the comparison of the corrosion rates between weight loss and polarization method with dependency on the KOH concentrations can also be found for highly doped Si wafers (dopant concentrations ~10^19^ 1/cm^3^) [110].

The difference between the corrosion rates obtained from different methods originates from the nature of the reaction mechanisms. While the polarization method takes only the electrochemical reactions into account, weight loss or etch depth method considers the both electrochemical and chemical reaction mechanisms. The quantitative difference on the corrosion rates suggests that the corrosion of Si in aqueous alkaline media involves both chemical and electrochemical mechanisms while former is being more dominant [110,138]. Although the net reactions of the mechanisms are identical, involvement of the electron transfer and the initiative step indicate the nature of the mechanism. Allongue et al. proposed that the reaction is chemical in nature if H_2_O attacks first, whereas electrochemical mechanism requires electron injection from adsorbed OH^-^ ions to Si [138]. Both reaction paths, on the other hand, result in the same end products (silicates) that are present as dissolved species in the solutions. Critical concentrations of such species should be avoided in order not to form very viscous gel-like solutions; for example, 4M silicate concentration in 5M KOH is reported as a saturation limit [52].

The influence of the Si corrosion on the OCV profiles of Si-air cells with 1M, 3M and 5M KOH are illustrated in Figure 11b. The cells initially provide OCPs around 1.4 V which are then stabilized at 1.38 V with 1M KOH and at 1.43 V with 3M and 5M KOH for at least 24 h. The Si in the cell with 5M KOH shows corrosion rates up to 1500 nm/h that is determined by the weight loss method. Such an experiment reveals two important phenomena: (i) the cell voltage is not influenced by the corrosion mechanisms even though high corrosion rates are present especially in highly concentrated KOH electrolytes, (ii) Si surface does not get passivated at least under OCV conditions since Si continues actively being corroded even in low KOH concentrations. Additionally, in a battery application, more appropriate electrode design would be with higher surface areas in form of porous electrodes. However, due to possessing high corrosion rates already with flat electrode surfaces, a straightforward utilization of porous Si electrodes efficiently in alkaline solutions is eminently challenging.

#### 2.2.3. Discharge Behavior of Si in Aqueous Alkaline Electrolyte

**Figure 12:**
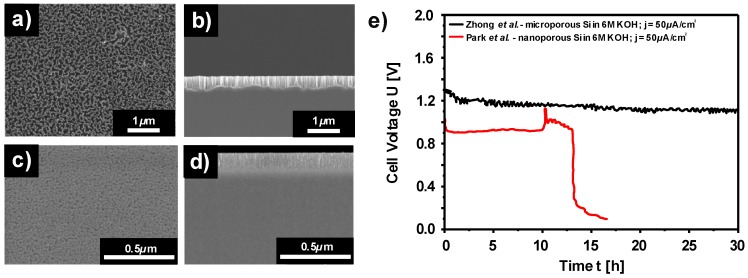
(**a**) Top-view of a Si surface after metal assisted chemical etching. (**b**) Cross-sectional view of a Si surface after metal assisted chemical etching (after Zhong et al.). (**c**) Top-view of an electrochemically modified Si surface in HF-based solutions. (**d**) Cross-sectional view of an electrochemically modified Si surface (after Park et al.). (**e**) Discharge profiles of Si-air cells employed with surface modified Si as anodes (after Zhong et al. and Park et al.). (Reprinted and adapted with permission from *ACS Appl. Mater. Interfaces* 7, 3126–3132, (2015). ©2015, American Chemical Society & *ChemSusChem* 5, 177–180, (2012). ©2015, Wiley & Sons) [127,128].

The first alkaline Si-air battery employing highly doped n-type nanostructured Si wafer was reported by Zhong et al. in 2012 [127]. The modification of Si by means of enhanced surface area was necessary since the unmodified flat Si surface could only be discharged for a short period of time (400 seconds). The limited discharge was attributed to Si surface passivation as a result of accumulation of the reaction products (Si(OH)_4_) on the surface. According to Zhong et al., the production rate of Si(OH)_4_ is slower than its dissolution rate on a flat Si surface; thus, the excess Si(OH)_4_ leads to the formation of SiO_2_ which terminates the discharge shortly. In order to overcome this issue, the surface area was considerably increased by a chemical surface modification method producing silicon nanowire bundles of up to 1.5 µm thickness on the Si surface [127,157]. As shown in Figure 12a,b, a microporous top layer was created as a result of metal-assisted chemical etching. By employing such modified Si as an anode electrode, the battery could be discharged at a voltage of 1.2 V with 50 µA/cm^2^ for at least up to 30 h (Figure 12e). The prolonged discharge time from 400 s (flat surface) to 30 h was attributed to roughened Si surface which ensures it to be active by effectively removing the discharge product Si(OH)_4_. Corrosion behavior of the surface modified Si in such an alkaline Si–air battery has been investigated by etch depth (step height) method. The Si wafers were discharged in KOH electrolytes with various concentrations for 7 h at a current density of 50 µA/cm^2^ or 100 µA/cm^2^. Accordingly, the step height differences between the reacted and non-reacted areas of the Si surface were obtained and the corresponding corrosion rates were calculated as 1.34 µm/h, 0.95 µm/h, and 0.24 µm/h for 6 M, 2 M, and 0.6M KOH solutions, respectively [127]. The high corrosion rates of Si in concentrated KOH electrolytes were reduced effectively by lowering the KOH concentration. Additionally, specific capacities up to 1206 mAh/g (0.6M KOH) were determined by considering the weight loss of Si (obtained from step height method).

In a follow up study, another alkaline Si-air battery with highly B-doped (p-type) Si electrode was reported by Park et al., who adapted slightly different approach [128]. Instead of using a chemical etching method for the surface modification, however, electrochemical etching in HF-based solution was performed. Employing an electrochemical method allowed the control on pore diameter and thickness during the formation of nanoporous Si structures. As an example, top view and cross-sectional view of the nanostructured Si electrodes are depicted in Figure 12c,d. According to the results on the influence of pore diameter and thickness on the discharge profiles of Si half-cell experiments (in 0.1M KOH at 5 µA/cm^2^), it was found out that Si anodes with thicker porous layer and smaller pore diameter provided better discharge performance. In a full-cell setup with 6M KOH electrolyte, on the other hand, such an optimized nanostructured Si electrode could be operated only up to 600 s at 10 µA/cm^2^. This issue was attributed to the mild anodization of the Si electrode during the surface modification by the electrochemical etching method. It was supported by XPS results that the SiO_2_ content was enriched upon the surface modification; therefore, high coverage of oxide in the nanopores lead to reduced discharge times. In order to overcome this, an extra oxide removal step by exposing Si to concentrated HF solution was employed after Si surface modification process. The discharge characteristic of such a Si electrode in 6M KOH is illustrated in Figure 12e. The discharge performance of the battery was improved from 600 s to 48000 s (~13 h) at a current density of 10 µA/cm^2^ with a stable discharge voltage around 0.9 V; in comparison to previous study, however, the discharge performance of the nanostructured Si electrodes was still lower.

**Figure 13:**
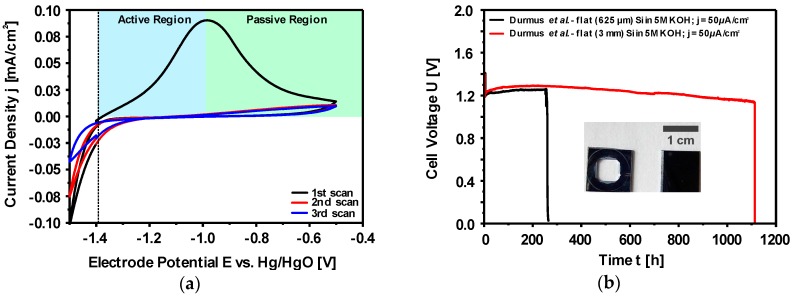
(**a**) Cyclic voltammogram of Si recorded at room temperature in 5M KOH solution. Light blue region as identified between OCP and passivation peak potential is defined as active region. Beyond the passivation peak potential is assigned to be passive region due to formation of passivation layer. (**b)** Long run discharge profiles of Si-air cells with different Si wafer thicknesses in 5M KOH at 25 °C with 50% relative humidity. Inset figure represents a photo of a fully discharged Si wafer in comparison to a fresh Si wafer (625 µm thick).

Up to this point, both studies reported the necessity of Si surface modification due to instant passivation of flat Si surface upon discharge. In a recent study this passivation phenomenon of polished (flat) Si wafer electrodes was investigated by Durmus et al. in cyclic voltammetry and galvanostatic discharge experiments [52]. The evidence of the surface passivation was obtained by cyclic voltammetry (CV) experiments, in which the highly As-doped <100> flat Si was cycled three times in 5M KOH. As depicted in Figure 13a, the cyclic voltammogram showed a single oxidation peak only in the 1^st^ scan; following cycles did not provide any anodic oxidation current due to the passive surface. Further investigations on the polished Si wafers with potential limited CV experiments revealed two different regions as active and passive (Figure 13a). In the active region (up to the passivation peak potential), the rate of oxide formation on the Si surface is lower than its dissolution rate; hence, Si actively dissolves in the electrolyte. For potentials more anodic than the passivation peak potential, the oxide dissolution rate cannot keep up with its formation rate; consequently, the anodic current decreases due to complete coverage of the surface by oxide layer. According to the results of CV experiments, Si surface is expected to remain active as long as the anodic potential does not exceed the passivation peak potential. This was confirmed by 24 h galvanostatic discharge experiments of Si-air batteries (with flat Si anode) under 50 µA/cm^2^ current densities. Contrary to the previous reports, the Si-air batteries with flat Si electrode could be discharged not only in 5M KOH but even in 0.75M KOH electrolytes (lower dissolution of reaction products in diluted KOH solutions) at a stable cell voltage above 1 V. The recovery of the cell voltage back to OCV upon discharge termination is also a clear indication of oxide-free surfaces. In this regard, these results showed an agreement with those from Palik et al. who reported that anodic potentials could be applied on Si electrode without passivating the surface for 24–48 h [134].

The high activity of Si in alkaline media in combination with non-passivated surfaces during discharge slightly alters the corrosion behavior of Si. In comparison to OCV corrosion, the Si surfaces are prone to enhanced corrosion under electrochemical discharge [52]. Therefore, following a 24 h discharge profile, the Si anodes could provide only ~3% mass utilization efficiencies, which indicate that 97% of the overall weight loss of Si material was consumed by the corrosion reaction. Under these conditions, enhancement of the surface area in form of porous electrode design remains as a formidable challenge since high surface area would favor the corrosion reactions in the battery. Up to 4M Si content in the electrolyte, nevertheless, the discharge profiles were not affected considerably; above this limit, viscous solutions were obtained. The corrosion reaction products were also analyzed by means of in-situ ^29^Si nuclear magnetic resonance (NMR) and formations of the silicates were identified by following the studies of Engelhardt et al. [158,159] and Harris et al. [160,161,162].

As a result of substantial corrosion reaction and open-cell geometry, water is consumed as well as the electrolyte is pushed away by H_2_ gas due to increased inner cell pressure. In order to overcome this discharge limitation, Durmus et al. reported a new cell setup which sustained the discharge for longer times by ensuring the electrolyte level to be constant in the cell. A proof of concept—the discharge of Si-air battery with flat Si electrode is limited only by the available anode mass—is established as shown in Figure 13b. Firstly, in the new cell setup a Si wafer (highly As-doped <100> oriented) with 625 µm thickness was employed which provided stable discharge voltages above 1.2 V up to 260 h. Next, a thicker Si wafer (3 mm) with the same specifications was used as an anode electrode in the battery setup. In this case, the discharge lasted about 1100 h while the cell voltage remained above 1.1 V before the discharge was terminated. In both cases, the discharge termination originated from the complete consumption of the active Si anode as evidenced by the inset figure (Figure 13b). The comparison of the 625 µm thick Si wafer before (right) and after (left) the discharge process reveals the actively consumed area on the surface. According to the weight loss analysis, the cells could provide specific capacities of 100 mAh/g, specific energies of 140 Wh/kg, and mass utilization efficiencies of ~3% [52].

### 2.3. Non-Aqueous Si-Air Cells

**Figure 14:**
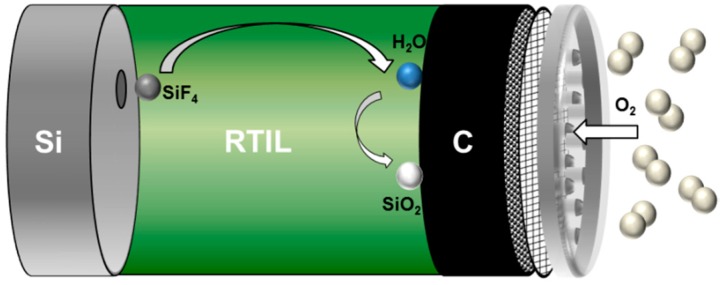
Schematic representation of general processes upon discharge of Si-air batteries with non-aqueous EMIm(HF)_2.3_F electrolyte.

The basic configuration of the non-aqueous primary Si-air cell, which is similar to that of the aqueous system, is depicted in Figure 14. It is composed of three main parts: silicon wafer as anode, room temperature ionic liquid (RTIL) as electrolyte, and carbon-based air electrode as cathode. As Si suffers from severe corrosion reaction in conventional alkaline electrolyte, one of the possible approaches to overcome this problem is focusing on ionic liquid electrolytes. In this regard, the RTIL EMIm(HF)_2.3_F is a potentially promising candidate which can improve the performance of Si-air batteries. The discharge of the cell results in the oxidation of Si to Si^4+^ (SiF_4_) at the anode while O_2_ from ambient atmosphere is being reduced at the air cathode yielding H_2_O and trihydrogenated fluoride anions. At the electrolyte-air electrode interface, the SiF_4_ produced during the anodic oxidation further reacts with H_2_O to form SiO_2_ deposits as end products. The electrochemical reactions upon discharge of a Si-air cell are proposed as [53]:

Anodic:         Si + 12(HF)_2_F^−^ ⇌ SiF_4_ + 8(HF)_3_F^−^ + 4e^−^ (6)

Cathodic:       O_2_ + 8(HF)_3_F^−^ + 4e^−^ ⇌ 2H_2_O + 16(HF)_2_F^−^ (7)

Formation of SiO_2_:  SiF_4_ + 2H_2_O + 4(HF)_2_F^−^ → SiO_2_ + 4(HF)_2_F^−^ (8)

Net reaction:           Si + O_2_ ⇌ SiO_2_       (9)

The standard half-cell potentials of anode and cathode have not been calculated by considering the thermodynamics to the best of our knowledge. Nevertheless, one can apply the Gibbs free energy formula (ΔG = −nEF) to the net reaction (Equation (9)) under the assumption of the formed SiO_2_ is alpha-quartz, which results in a theoretical cell voltage of 2.21 V. In practical cell application, on the other hand, the open circuit voltage can reach only up to 1.6 V [53,86].

In comparison to aqueous alkaline system, the reaction mechanisms are quite different and according to the proposed mechanisms, the air electrode plays a major role as the oxygen reduction as well as the end product (SiO_2_) formation takes place there. With respect to the reversibility of the system, the conditions for an electrochemical reduction of SiO_2_ are not yet clear especially in the ionic liquid electrolyte. Therefore, up to this point, the reported non-aqueous Si-air batteries are only primary systems.

#### Electrochemical Characteristics of Silicon Electrodes

The first studies on the formation of porous Si by electrochemical oxidation of low to medium doped Si wafers in room temperature ionic liquid (EMIm(HF)_2.3_F) already provided some hints about this electrolyte as a potential candidate for a possible battery application [163,164]. By pursuing this idea, Ein-Eli et al. investigated the behavior of medium and heavily doped Si wafers as well as an air electrode for the oxygen reduction in the same electrolyte [123]. For the battery operation, doping of the Si wafers is important in terms of conductivity, as Si is a semiconductor with low electronic conductivity. Basing on the promising results such as low corrosion rates and an average working potential of 1.0–1.2 V under relatively high current densities of up to 300 µA/cm^2^, the new concept Si-air was introduced [123]. Further studies by Cohn et al. led to new insights into the understanding of the Si-air battery behavior [53,140,142,143,144]. More recently, Aslanbas et al. also reported the effect of alloying of Si and Al on the discharge and corrosion behavior of cells with EMIm(HF)_2.3_F [165]. A detailed analysis of the non-aqueous Si-air batteries is covered within a review on non-aqueous non-alkali (NANA) metal-air batteries [50].

**Figure 15:**
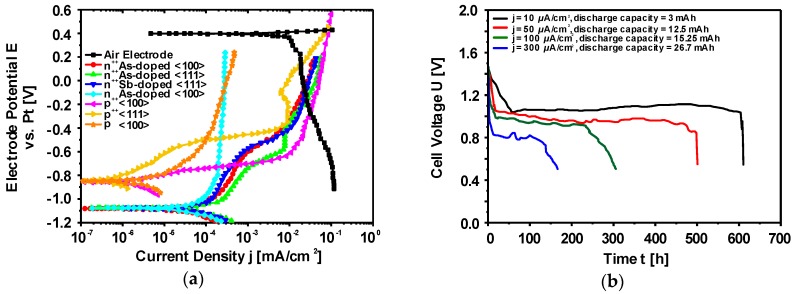
(**a**) Potentiodynamic polarization curves of As-, Sb-, and B-doped <100> and <111> oriented Si wafers and air cathode in EMIm(HF)_2.3_F solution (after Cohn et al.). (**b**) Discharge profiles of Si-air cells with As-doped <100> oriented Si wafers as anodes at different constant current densities (after Cohn et al.). (Reprinted and adapted with permission from *J. Power Sources* 195, 4963–4970, (2010). ©2010, Elsevier) [53].

Single crystal Si wafers with different specifications (crystal orientation, dopant type, dopant concentration) were investigated by potentiodynamic polarization experiments as an initial step (Figure 15a). According to the comparison of the potentials within the low current density region (< 1 mA/cm^2^) as well as the corrosion rates of individual Si wafers, it was decided to continue studying highly As doped <100> oriented Si (corrosion rate < 0.08 nm/min) in the Si-air batteries [53]. Typical discharge curves of the batteries are depicted in Figure 15b. The cell voltages are between 0.8–1.1 V under discharge current densities of 300–10 µA/cm^2^ [53]. The cells could be operated for relatively long time resulting in up to 26.7 mAh discharge capacities (<300 µA/cm^2^). From the X-ray photoelectron spectroscopy (XPS) analysis, an origin of the discharge termination is considered to be the SiO_2_ deposits (discharge products) on the cathode which lead to pore clogging in the air electrode in a similar way with Li-O_2_ batteries [53,166,167].

The impact of water on the discharge performance of Si-air batteries has been investigated in another study by Cohn et al. [142]. Since it was proposed previously that water involves in the SiO_2_ formation at the air cathode, addition of water into the hydrophilic EMIm(HF)_2.3_F electrolyte could influence the reaction zone and prolong the discharge. Under the discharge current density of 300 µA/cm^2^, introduction of 15 vol.% water in the electrolyte resulted in an increase of capacity by 35% in comparison to neat electrolyte [142]. The reason was attributed to shift of the SiO_2_ production zone away from the air cathode which prevents pore clogging and loss of active catalytic sites.

**Figure 16:**
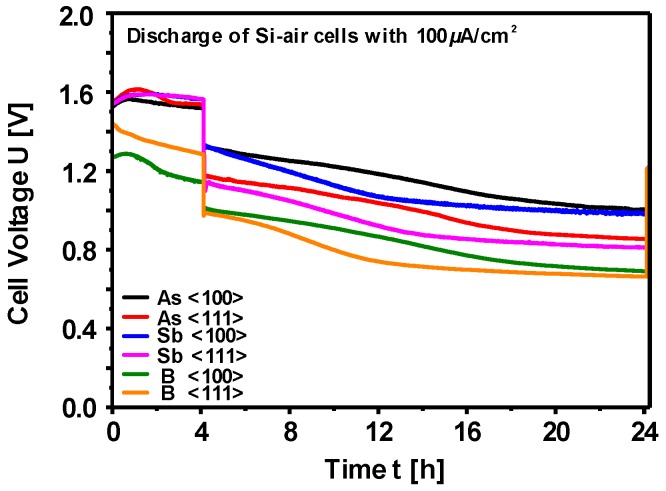
Open circuit voltage (OCV) and discharge profiles of Si-air cells with As-, Sb-, and B-doped Si anodes under constant current density of 100 µA/cm^2^ over 24 h.

**Figure 17:**
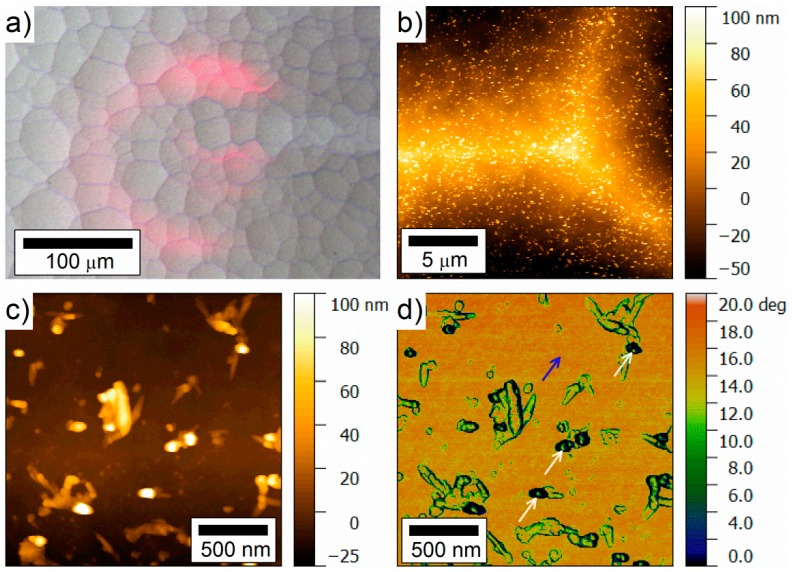
(**a**) Optical microscopy image of the discharged As <111> Si surface with 100 µA/cm^2^. (**b**) Low magnification atomic force microscopy (AFM) image of a wall/boundary of polygon structures. (**c**) High magnification AFM image. (**d**) Simultaneously recorded phase image of the high magnification AFM image. Blue and white arrows indicate the Si surface and the top of the particles, respectively. (Reproduced with permission from *J. Electrochem. Soc.*, 164, A2310–A2320 (2017). ©2003, The Electrochemical Society) [86].

The discharge termination mechanism was further analyzed by Jakes et al., who performed electron magnetic resonance spectroscopy (EPR) and XPS on the air cathode [143]. In addition to pore clogging by SiO_2_ reaction products, the results obtained by EPR and XPS on the air electrode after cell discharge revealed another mechanism for discharge termination; modification of the catalyst (MnO_2_) in the air cathode. According to proposed model the chemical structure of the MnO_2_ catalyst is converted to MnF_2_ upon discharge; hence, active sites for O_2_ reduction were lost. The conversion mechanism involves formation of H_2_O as a reaction product, which makes a positive impact by retarding this catalyst conversion mechanism significantly. Silicon/electrolyte and air cathode/electrolyte interfaces during the battery operation were characterized by electrochemical impedance spectroscopy (EIS) in order to gain further insights into discharge behavior of Si-air batteries [144]. Although the discharge failure mechanism was always attributed to the air cathode side due to pore clogging and catalyst conversion, EIS results showed increased impedance originating from the Si anode electrode during discharge. The impedance data was fitted by an equivalent circuit, in which the parameters related to space charge layer (capacitance and charge transfer) as well as to submicron pores (resistance and capacitance) were analyzed. Further investigations were performed by comparing the influence of the individual electrodes on the discharge profiles by half-cell experiments. The discharge potential and capacity of the battery were mainly dominated by the behavior of Si electrode. In a full-cell setup, while maintaining the same cathode the cells could be reactivated by replacement of the Si anode at the end of discharge [144]. Furthermore, in a recent study Durmus et al. have focused on the influence of Si wafer types on the electrochemical performance of Si-air batteries [86]. Anodes prepared from <100> and <111> oriented Si wafers doped with As, Sb, or B have been investigated by galvanostatic discharge experiments in full-cells over 24 h. The typical discharge profiles under 100 µA/cm^2^ discharge current density are depicted in Figure 16. Evaluation of the individual Si wafer types were performed with respect to (i) discharge voltage characteristics at different current densities, (ii) corrosion rates and anode mass conversion efficiencies, and (iii) specific discharge energies. For all types of Si anodes, the comparison of the corrosion rates calculated from weight loss and potentiodynamic polarization experiments revealed two orders of magnitude difference, which indicates a chemical corrosion contribution like in the aqueous alkaline systems while being at a much slower rate. Therefore, an overall assessment of the corrosion rates of Si in EMIm(HF)_2.3_F electrolyte has to be based on weight loss measurements. The suitability of this method requires that no deposits are present on the surface. This was further confirmed by atomic force microscopy (AFM) images as shown in Figure 17. The typical polygon-like surface morphologies of As <111> Si wafer (after discharge with 100 µA/cm^2^) were analyzed by AFM. The walls of polygon-like structures (Figure 17a) consist of numerous particles which correspond to pillars on the surface (Figure 17b and c). In the phase signal image (Figure 17d) at high magnification, a contrast was only obtained at the edges of the particles and not between the surface (blue arrow) and the top part (white arrows) of the particles; hence, no deposits are present on the Si surface after the cell operations. According to the overall ranking of the Si wafer types based on the battery parameters, As <111> Si wafer was considered to be the best suitable anode material for Si-air batteries with EMIm(HF)_2.3_F electrolyte. The cells provide specific energies of more than 1600 Wh/kg (related to anode weight loss) when operated at 100 µA/cm^2^ (see Table 4).

## 3. Iron-Air Batteries

### 3.1. Overview

**Figure 18:**
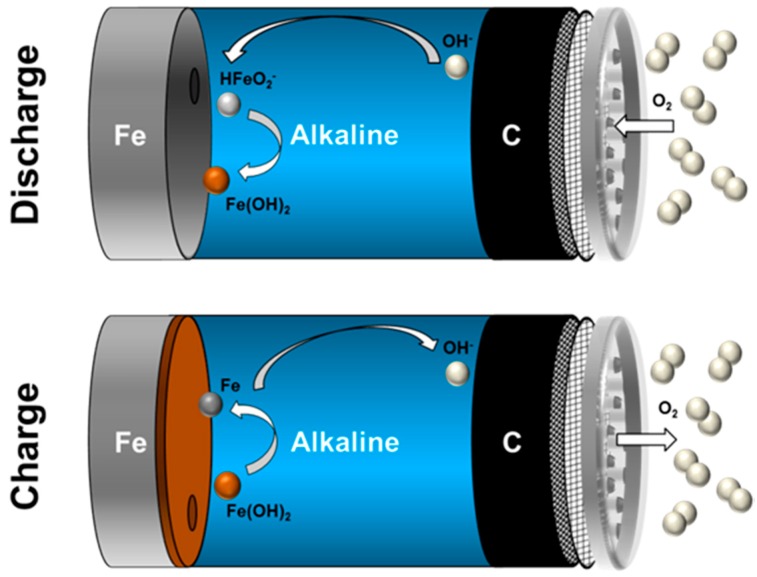
Schematic representation of a rechargeable alkaline Fe-air battery showing the general processes occurring upon charge and discharge of the battery.

In the most general case, schematically depicted in Figure 18, Fe-air batteries consist of a ferrous (mostly iron) anode and a (carbonaceous) air cathode immersed into a liquid aqueous electrolyte at room temperature. During the operation of the cell, the two solid electrodes provide the oxidation of Fe and the reduction of O_2_ during the discharge- as well as the opposite reactions during the potential recharge [43,119,168]. In secondary Fe-air batteries, typically, concentrated alkaline solutions like 6M KOH are used as electrolyte, facilitating the sluggish oxygen reduction and oxygen evolution reaction (ORR/OER) on the air electrode, while not being too corrosive to the iron anode [37,96,169]. In concentrated alkaline electrolytes, most favorably, the following two reactions occur on the individual electrodes during charge- (←) and discharge (→) of the battery [170,171]:

Iron Electrode:  Fe + 2OH^−^ ⇌ Fe(OH)_2_ + 2e^−^      E^0^ = −0.88 V (10)

Air Electrode:   ½ O_2_ + H_2_O + 2e^−^ ⇌ 2OH^−^      E^0^ = 0.40 V (11)

Cell Reaction:     Fe + ½ O_2_ + H_2_O ⇌ Fe(OH)_2_   U = 1.28 V (12)

As a result of the underlying reactions, Fe-air batteries provide a theoretical cell voltage of U = 1.28 V and a specific energy of 764 Wh/kg (including oxygen uptake), based on the weight of the primary discharge product (Equation (10)) [44]. Moreover, beyond the oxidation of Fe to Fe(OH)_2_, the iron electrode may be discharged further, according to the following reactions [172]:

Deep Discharge:   Fe(OH)_2_ + OH^−^ ⇌ FeOOH + H_2_O + e^−^  E^0^ = −0.56 V (13)

Deep Discharge:  3Fe(OH)_2_ + 2OH^−^ ⇌ Fe_3_O_4_ + 4H_2_O + 2e^−^  E^0^ = −0.66 V (14)

However, despite the higher specific energy (913 Wh/kg), the oxidation of Fe(OH)_2_ to FeOOH or Fe_3_O_4_ is not preferred, since Fe(III)-species appear to be more stable and are, therefore, prone to incomplete reduction during the recharge. Furthermore, the reactions given in Equations (13) and (14) provide lower electrode potentials compared to the primary reaction (Equation (10)), which makes the former comparatively unattractive for practical application [43,173]. Hence, most researchers typically abort the discharge of Fe-air batteries once the available iron has been oxidized to Fe(OH)_2_ completely, considering the reactions according to Equations (13) and (14) as a deep-discharge reserve [173,174].

During the discharge of an Fe-air battery, the primary reaction product of the anode, Fe(OH)_2_, accumulates on the iron electrode surface, driven by the comparatively low solubility of the discharge products in alkaline electrolyte, which is both bane and boon for the electrochemistry of iron electrodes. On the one hand, Fe(OH)_2_ is an electronically insulating species that prevents the extensive utilization of the anode material by the formation of a passivating layer on the iron electrode surface [175,176,177]. On the other hand, the limited solubility of the discharge products fosters the uniform deposition of Fe(OH)_2_ on the iron anode, which, in turn, prevents unfortunate dendrite formation and an extensive macroscopic shape-change of the anode material upon repeated cycling [178,179,180,181,182].

According to the solubility-driven formation of the passivating layer, the discharge performance of Fe-air batteries is clearly limited by the surface area rather than the total amount of the anode material, which is a specific but no detrimental issue for Fe-air-compared to other MAB systems [36,40,183]. In case of Si-air batteries, the discharge capacity of the cell is solely limited by the bare amount of available silicon given the extensive solubility of the discharge products in alkaline electrolyte [52]. In case of Li-O_2_ batteries, the discharge products accumulate on the carbonaceous cathode, limiting the discharge performance by the access of oxygen to the air cathode rather than the anode properties [40]. However, in particular contrast to Zn-air batteries, dendrite formation and a macroscopic shape-change of the anode have, alternatively, also rarely been reported for Fe-air batteries, boosting the safety and the reversibility of the system, especially at 100% depth-of-discharge (DoD) of the iron electrode [173,174,184].

Beyond the challenging passivation behavior of iron in alkaline electrolyte, a second major issue for the yet unresolved implementation of highly-efficient secondary Fe-air batteries lies in the obstinate reduction of Fe(OH)_2_ back to metallic iron (Fe). Driven by a comparatively high overpotential for the recharge, the reduction of Fe(OH)_2_ to Fe competes with the hydrogen evolution reaction (HER) according to Equation (15) [185,186,187], which has a twofold effect for the system: First, extensive HER may result in a pronounced loss of water, which might cause the battery to dry-up and fail if the water content is not controlled. Second, the HER significantly decreases the coulombic efficiency of the cell, as some of the applied charge is diverted in the unintended side-reaction, which should clearly be omitted by appropriate strategies [93,188,189]:

Hydrogen Evolution Reaction:  H_2_O + e^−^ ⇌ ½ H_2_ + OH^−^  E^0^ = −0.83 V (15)

Furthermore, other research topics regarding Fe-air batteries aim at the reduction of the self-discharge (i.e., the corrosion) of iron in alkaline electrolyte, the enhancement of the electrochemically reversibility of the solid discharge products and the general improvement of the air electrode performance [37,100,182,190].

### 3.2. Thermodynamics of Iron in Aqueous Electrolyte

**Figure 19:**
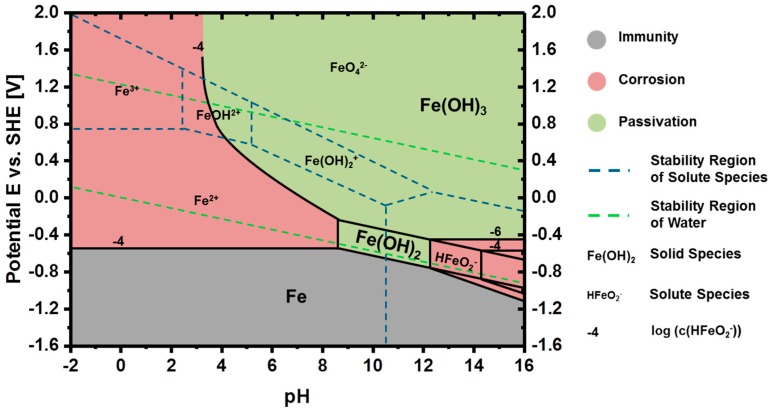
Pourbaix diagram of iron considering Fe(OH)_2_ and Fe(OH)_3_ as solid substances only. (Redrawn from [193].).

Fe-air batteries typically employ liquid aqueous electrolytes such as 6M KOH, owing to their excellent electrochemical activity and ionic conductivity [96,191]. Given the use of aqueous electrolytes, classic (aqueous) thermodynamics applies to the fundamental description of Fe-air cells, which is helpful in order to grasp superordinate correlations regarding the general battery chemistry. Representing the thermodynamics in aqueous media, the Pourbaix diagram of iron shown in Figure 19 displays stability regions and electrochemical reactions for important ferrous species depending on the electrode potential and the pH of the electrolyte [147,192].

From a general perspective, the Pourbaix diagram identifies three regimes of interest for the reactivity of iron electrodes in aqueous electrolyte, namely the “immunity”-, the “passivation”—and the “corrosion”—regime [193]. Within the “immunity”—regime (grey), bare iron is stable against any (electro-)chemical reaction. In the ‘passivation’—regime (green), iron directly reacts with the electrolyte forming a thin Fe(OH)_2_-layer on the iron electrode surface that prevents further reactions of the metal beneath the passive film. Furthermore, the “corrosion”-regime (red) is separated in two regions for either acidic (pH < 8.3) or concentrated alkaline solutions (pH > 12). Within these regions, iron corrodes spontaneously until the corrosion ceases by the eventual formation of a protective layer, which has already been investigated by Michael Faraday and other researchers as early as 1790 [194].

**Figure A1:**
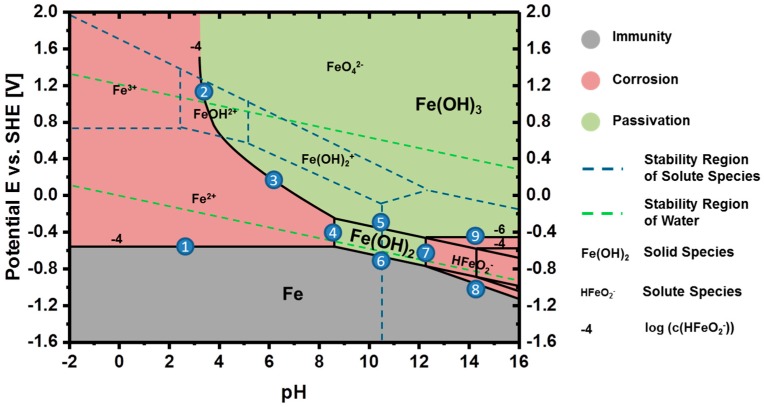
Pourbaix diagram of iron including the transition reactions (1–9) from one considered species to the other. (Redrawn from Pourbaix M (1974) Iron. In: Atlas of Electrochemical Equilibria in Aqueous Solution, 2nd ed. NACE-International, Houston, pp. 307–321.).

According to the fundamental meaning of thermodynamics, Pourbaix diagrams provide a first good idea about the reactivity of a metal in aqueous media. Each line in a Pourbaix diagram represents one particular reaction that occurs at a given pH and a given electrode potential, depending on the concentration of the, possibly, involved solute species (see also Figure A1 and Table A1 in Appendix A) [193]. However, Pourbaix diagrams do not state the conditions for the displayed reactions. Specific information about reaction rates, overpotentials or the continuance of the reaction products must be acquired by experimental techniques like cyclic voltammetry, open-circuit potential transient measurements, or galvanostatic charge-/discharge experiments for every electrode [192,195,196].

### 3.3. Discharge vs. Corrosion of Iron

The Pourbaix diagram in Figure 19 identifies corrosion as a potential issue for Fe-air batteries that employ concentrated alkaline electrolytes. Above pH = 12 and at a HFeO_2_^−^-concentration of c(HFeO_2_^−^) = 10^−6^ M, iron is thermodynamically unstable at an electrode potential above *E* = −0.8 V vs. SHE (*E* = −0.9 V vs. Hg/HgO) and is due to corrode with a decreasing lower potential limit depending on the pH of the electrolyte [193]. However, the latter does not necessarily prohibit the application of iron as an electrode material for batteries, considering the duality of corrosion and the discharge reaction in alkaline electrolyte [171].

**Figure 20:**
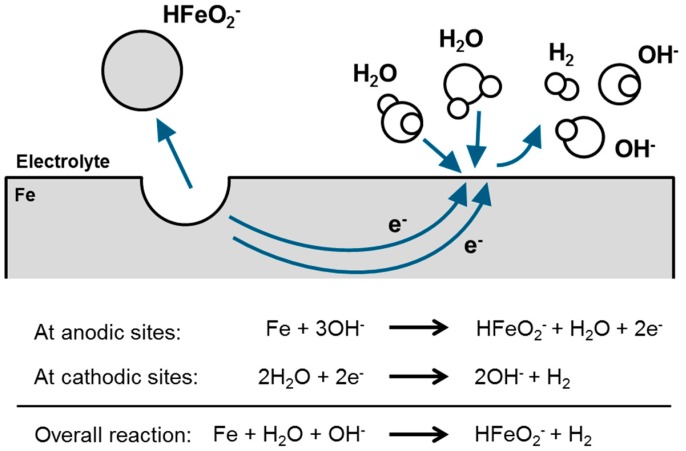
Corrosion of iron in deaerated alkaline electrolyte. (Adapted from [197].).

According to the most fundamental definition, corrosion is the destructive attack of a metal due to its reaction with the environment [197]. More specifically, corrosion is also defined as an electrochemical process that occurs not by direct chemical reaction of a metal with its environment, but rather through the operation of coupled half-cell reactions *on the same* metal surface, mostly accompanied by the evolution of hydrogen as schematically depicted in Figure 20 [197]. In contrast to that, the discharge of iron in an iron-based battery can be defined as the reaction of the iron electrode with the environment supported by a coupled half-cell reaction *located on a second*, electrically connected electrode [198]. However, although potentially different in the exact mechanism, both reactions formally result in the same reaction product, i.e., FeOH_2_, given by Equations (16) and (17) [119,177,190,197,199]:

Charge-/Discharge Reaction:    Fe + 2OH^−^ ⇌ Fe(OH)_2_ + 2e^−^    E^0^ = −0.88 V (16)

Corrosion Reaction:      Fe + 2H_2_O → Fe(OH)_2_ + H_2_      spontaneous (17)

Following the definitions for corrosion and the discharge reaction, the difference between both processes is merely a question where the electrons released during the oxidation of iron are transferred to. In case of the discharge reaction, the released electrons are transferred to the counter electrode via an outer circuit, which is useful for practical application. In case of corrosion, the released electrons are dissipated by spontaneous hydrogen evolution or other processes on the same surface, impairing the material and decreasing the efficiency of a battery [181,188]. Which electrode reaction dominates the other in the individual case depends on the reaction kinetics under the given experimental conditions [171]. “Corrosion”-conditions, identified in the Pourbaix diagram, do not necessarily prohibit Fe-air battery operation, while even acidic Fe-air batteries have been reported in literature [200]. The “corrosion”-regime in the Pourbaix diagram rather identifies the conditions upon which corrosion may be present and corrosion inhibiting strategies should be considered. The lower the corrosion rate, the higher is the conversion efficiency and the shelf-life of a battery in the charged state [181]. The latter is especially obvious when comparing the performance of alkaline Si- and Fe-air batteries. Since the actual corrosion of iron in concentrated alkaline electrolyte is relatively low compared to the corrosion of silicon, the electrochemical characteristics of iron make the application of alkaline Fe-air batteries currently significantly more efficient than the application of alkaline Si-air batteries [52,174].

Given the formal identity of the discharge- and the corrosion product in alkaline electrolyte (Equations (16) and (17)), the corrosion of iron is typically investigated by two sorts of experiments, i.e., potentiostatic and potentiodynamic measurements, which may further be subdivided into in-situ and ex-situ experiments, depending on the actual investigation [201]. The difference between the two sorts of experiments consists in the approach of how the oxidation of the investigated electrode is executed. In a potentiostatic corrosion experiment, the sample is left under preset environmental conditions and is investigated during or after a certain period of time, without the external adjustment of the electrode potential. In contrast to that, in a potentiodynamic corrosion experiment, the iron electrode is investigated after or during the electrochemical cycling between the ‘immunity’—and the ‘corrosion’—regime (Equation (16), implicitly assuming that the structure and the composition of the reaction product is identical to the product of the spontaneous corrosion, although the “forced” oxidation resembles a discharge [202,203,204,205,206]).

**Figure 21:**
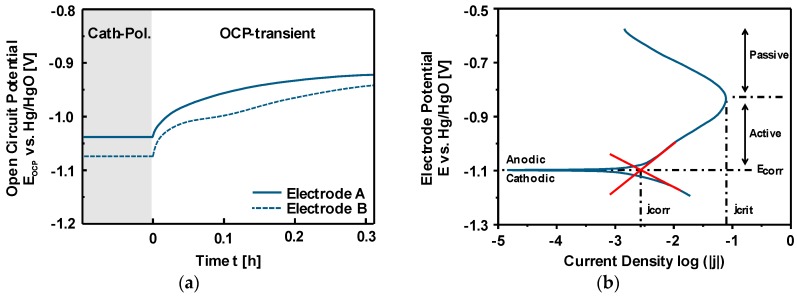
Passivity and corrosion of iron. (**a**) Open-circuit potential transient measurements for the investigation of electrode kinetics and the corrosion behavior of iron in alkaline electrolyte depending on the electrode composition. (Adapted from [195].) (**b**) Potentiodynamic polarization curve for iron in concentrated alkaline electrolyte identifying the active and passive region, the critical current density (j_crit_) as well as the corrosion potential (E_corr_) and the corrosion current density (j_corr_).

Among the numerous experimental techniques in corrosion research [201], two major methods that have been developed over the past decades are namely open-circuit potential (OCP-)transient [195,207] and electrode polarization measurements [196,208], which are an example for a potentiostatic and a potentiodynamic corrosion experiment, respectively. Figure 21a displays the potential relaxation behavior of two iron electrodes after extended cathodic polarization in alkaline electrolyte during an OCP-transient measurement. Due to the initial cathodic polarization, the iron electrodes have electrochemically been reduced for a certain period of time and were then left for OCP-recovery, which occurs due to interfacial reactions between electrode and electrolyte, e.g., via the equilibration of the electrode reactions or the build-up of pseudo-capacitances due to the adsorption of solute species in the electrolyte [195]. In case of the example in Figure 21a, given the analogous pretreatment, the OCP-transients of Electrode A and B appear to be similar on a broad scale, but exhibit detailed differences, which depend on the corrosion behavior of the electrode material. Driven by the slightly different electrode composition, in this case, the potential decay of Electrode B starts at higher potentials vs. Hg/HgO and appears to take much longer than for Electrode A, implying increased passivity of Electrode B compared to Electrode A. Beyond the electrode composition, other important parameters for the corrosion behavior are given by the pH and the composition of the electrolyte as well as the pretreatment characteristics of the electrode such as the cathodic polarization time and the reduction current density, which may be varied to determine qualitative material properties [195,209,210,211]. Furthermore, it was shown by Vijayamohanan et al. that the OCP-decay may also be used as a measure for the state-of-charge (SOC) of battery electrodes, since the corrosion depends on the available active surface area, which is diminished depending on the galvanostatic discharge of an iron electrode [181,195]. However, the implicit difficulty of a reproducible initial state complicates the quantitative analysis of practical electrodes, especially in complex electrolyte systems [201].

Figure 21b shows a schematic potentiodynamic polarization curve for iron in concentrated alkaline electrolyte, which is the characteristic result of a polarization measurement. Due to the potential sweep during the measurement, the potential of the iron electrode was driven from the ‘immunity’-regime below −1.1 V vs. Hg/HgO towards the ‘corrosion’-regime in the Pourbaix diagram, resulting in a steeply increasing current density in the anodic branch of the curve, which agrees with the increasing reaction rates for the dissolution of iron in the “Active Region” [212]. However, since the reaction rate of a metal is not unlimited, at some point, depending on the actual material, a further increase in the electrode potential culminates in a current maximum; the critical current density for the oxidation of the electrode material (*j_crit_*). The critical current density marks the transition from the “Active” towards the “Passive Region”, which is a consequence of the continuous precipitation of the oxidation products on the iron electrode. The critical current density provides a measure for the maximum current that may be provided by an iron electrode prior to its electrochemical passivation. The latter displays an important electrode characteristic for Fe-air batteries [100,177,213]. In the interest of a high performance material, the critical current density should be as high as possible, while the transition from the “Active”—into the “Passive”—region should occur at comparatively low potentials, aiming at an enhanced power density [212]. Furthermore, from a Tafel-fit around the transition point from the anodic to the cathodic branch of the polarization curve, electrode specific corrosion properties may be derived. As schematically depicted in Figure 21b, the intersection of two tangents in the Tafel-region provide the electrode corrosion potential E_corr_ as well as the corrosion current density j_corr_, which may be understood as benchmarks for the comparison of different samples [201]. In the interest of a highly efficient electrode, the corrosion current of the iron anode, tantamount to the amount of corrosively consumed active material on standby, should be as low as possible, while the corrosion potential should be as high as possible in order to omit the competition between the Fe(OH)_2_-reduction and the HER during the recharge of the electrode.

### 3.4. Charge-/Discharge Characteristics of Ferrous Electrodes

Electrochemical cycling of ferrous electrodes in Fe-air batteries typically involves the utilization of the Fe/Fe(II)- and, possibly, the utilization of the Fe(II)/Fe(III)-redox-couple in a consecutive manner, if the full range of the applicable electrode potential is considered [43,214,215]. Analyzing the relation of the redox-reactions, mostly, the potentiodynamic behavior of the applied material is investigated by cyclic voltammetry [216]. Depending on the material, the cyclic voltammogram (CV) of ferrous electrodes shows several peaks, which refer to distinct redox-reactions on the electrode surface, but may be difficult to assign without additional chemical analyzes [217]. In fact, the electrochemical properties, e.g., reaction kinetics, overpotentials and corrosion behavior, of iron depend on various parameters, which might be inconspicuous at a first glance like the purity of the electrode material [173,218], but may occasionally impede the comparability of different studies due to the appearance or suppression of individual reaction processes [203,215,219].

**Figure 22:**
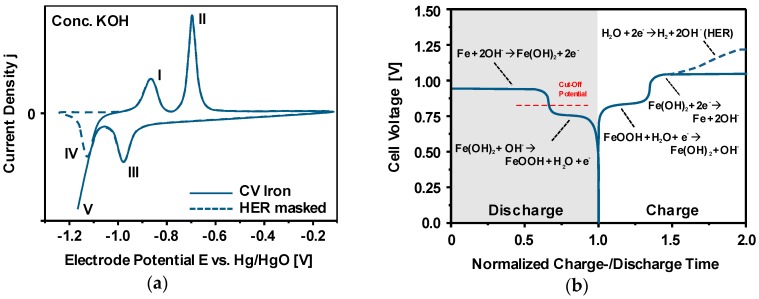
Charge-/discharge properties of iron in alkaline electrolyte. (**a**) Ideal cyclic voltammogram of iron in concentrated alkaline electrolyte. (Dashed blue line indicates the competitive recharge reaction (IV) with the hydrogen evolution reaction (HER).) (**b**) Idealized charge-/discharge profile of an Fe-air full-cell using 6M KOH as electrolyte. (Dashed red line indicates a suitable cut-off potential avoiding the formation of barely reversible Fe(III)-species.).

In the least complicated case, the CV of iron in concentrated alkaline electrolyte shows two distinct oxidation and two distinct reduction peaks within a potential range from −1.3 V to −0.3 V vs. Hg/HgO, as schematically depicted in Figure 22a [220,221]. Starting in the reduced state at −1.3 V, the two oxidation peaks refer to the oxidation of Fe to Fe(OH)_2_ according to Equation (10) (Peak I) [220,222] and the subsequent oxidation of the resulting Fe(OH)_2_ to FeOOH [204,205] or Fe_3_O_4_ [171,223] according to Equation (13) or Equation (14) (Peak II). Conversely, the reduction reactions corresponding to Peak III and Peak IV refer to the reversal of the reactions assigned to Peak II and Peak I, i.e., the reduction of FeOOH or Fe_3_O_4_ to Fe(OH)_2_ and Fe(OH)_2_ to Fe [171,220,222]. Furthermore, given the definite electron ratio of Equation (10) vs. Equations (13) and (14), Peaks I & II and Peaks IV and III display an intensity ratio of 2:1, as expected from theoretical considerations.

However, in many cases, the electrochemistry of iron does not appear as simple as described above and provides much higher complexity than the CV shown in Figure 22a. With respect to this, first of all, the occurrence of the HER has to be considered as an additional reaction, which has frequently been reported to impede the rechargeability of iron electrodes. Depending on the material-specific overpotential, the HER on iron (Equation (15)) occurs at a potential between −1.00 V and −1.20 V vs. Hg/HgO [211,215]. Due to the occurrence of the HER, mostly, the reduction of Fe(OH)_2_ to Fe is concealed by a drastically increasing cathodic current, indicated by the onset of Peak V in Figure 22a. As a consequence of the similar redox-potentials for the recharge of the iron electrode and the electrolysis of water, the reduction of Fe(OH)_2_ competes with the HER resulting in an overlap and the indistinguishability of both processes in the CV [181,182,224].

Moreover, with respect to the anodic polarization of the iron electrode, also even more than just two oxidation peaks have been reported by several researchers [203,215,225]. Haupt et al., for example, reported the observation of four rather than two oxidation peaks for an iron sheet in 1M NaOH at about −0.80 V, −0.60 V, −0.45 V and −0.20 V vs. SHE. Based on a dedicated analysis of the CV, they attributed these peaks to the oxidation of (i) adsorbed hydrogen, (ii) iron to iron(II) hydroxide, (iii) iron(II) oxide or hydroxide to iron(III) oxide, and (iv) an inner iron(II) oxide layer to iron(III) oxide [203]. In contrast to that, Schrebler–Guzmán et al. reported the observation of three oxidation peaks at about −0.8 V, −0.6 V and −0.4 V vs. SCE in 1M KOH solution. In this case, the authors attributed the redox-reaction peaks to a two-step oxidation of Fe to Fe(OH)_2_ (Peaks I and II) and a one-step oxidation of Fe(OH)_2_ to FeOOH (Peak III), which is a clear difference compared to the conclusions by Haupt et al. [215]. Furthermore, it has been shown by Andersson et al. that the occurrence of individual peaks in the CV is also clearly temperature dependent, emphasizing the importance of the experimental conditions for the electrochemical investigation [222]. At room temperature, Andersson et al. found two oxidation peaks for a porous iron electrode in 4.5M KOH corresponding to the consecutive oxidation of Fe to Fe(OH)_2_ and Fe(OH)_2_ to FeOOH, whereas at 50°C and higher they observed three peaks corresponding to the reactions at room temperature as well as the direct oxidation of Fe to FeOOH:

Direct Oxidation:  Fe + 3OH^−^ ⇌ FeOOH + H_2_O +3e^−^  E^0^ = −0.65 V (18)

Beyond bare iron, the application of ferrous materials like steel and iron oxides have also frequently been investigated by several researchers [185,226,227,228]. Among them, Yamamoto investigated the reactivity of mild steel in concentrated alkaline electrolyte with and without the previous activation in 1M KCl solution and found the discharge current densities of the electrode to be significantly improved due to the applied pretreatment [229]. Furthermore, in several articles about the impact of carbon materials on the charge/discharge performance of iron oxides, Hang et al. pointed out that the exact electrode composition is important for its electrochemical behavior and may be tailored in order to achieve superior electrode performances. Depending on the amount and the electrical connection of the applied iron oxide to carbon, i.e., depending on the conductivity of the electrode, Hang et al. observed varying numbers of peaks as well as different shapes of the individual CVs, which obviously affects the charge-/discharge behavior during galvanostatic cycling as well [172,187,230].

Targeting the implementation of electrochemically rechargeable Fe-air batteries, beyond cyclic voltammetry, galvanostatic charge-/discharge experiments are a second major method for the investigation of characteristic electrode properties, providing quantitative information with respect to the electrode capacity [16,25,56,57]. In the battery application-near method, the reactions occurring on the investigated electrodes are represented by voltage plateaus as a function of time, which is schematically depicted in Figure 22b [168]. The length of each plateau depends on the amount of charge provided by- or required for the corresponding reaction. In the most general case of a bare iron electrode in alkaline electrolyte, directly equivalent to the observations in the CV in Figure 22a, the galvanostatic charge-/discharge profiles show two plateaus, which refer to the oxidation of Fe to Fe(OH)_2_ and Fe(OH)_2_ to FeOOH/Fe_3_O_4_ and vice versa [222,231,232]. Using the galvanostatic method, especially the coulombic efficiency of the investigated electrode may easily be derived by a direct comparison of the applied charge- and the resulting discharge capacity [96,173,233]. In addition, the loss of electric charge due to the HER during the recharge step is directly accessible by the deviation of the upper charging plateau from a horizontal line, indicated by the dashed blue line in Figure 22b [171]. The higher the tendency to hydrogen evolution during the recharge, the shorter will be the charging plateau for the reduction of Fe(OH)_2_ [177]. Furthermore, due to the sharp division of the discharge plateaus, during galvanostatic cycling, the limitation of the charge-/discharge reaction to a certain discharge product is comparatively simple, which is advantageous for the repeated electrochemical cycling of the battery. Fe(III)-species appear electrochemically more stable than Fe(II)-species and are, therefore, prone to incomplete and even irreversible oxidation during the recharge. The latter should be prevented by a cut-off potential in between the discharge plateaus in order to increase the reversibility of the iron electrode (cf. Figure 22b) [93,173,218].

### 3.5. First Oxidation Mechanism of Iron in Alkaline Electrolyte

**Figure 23:**
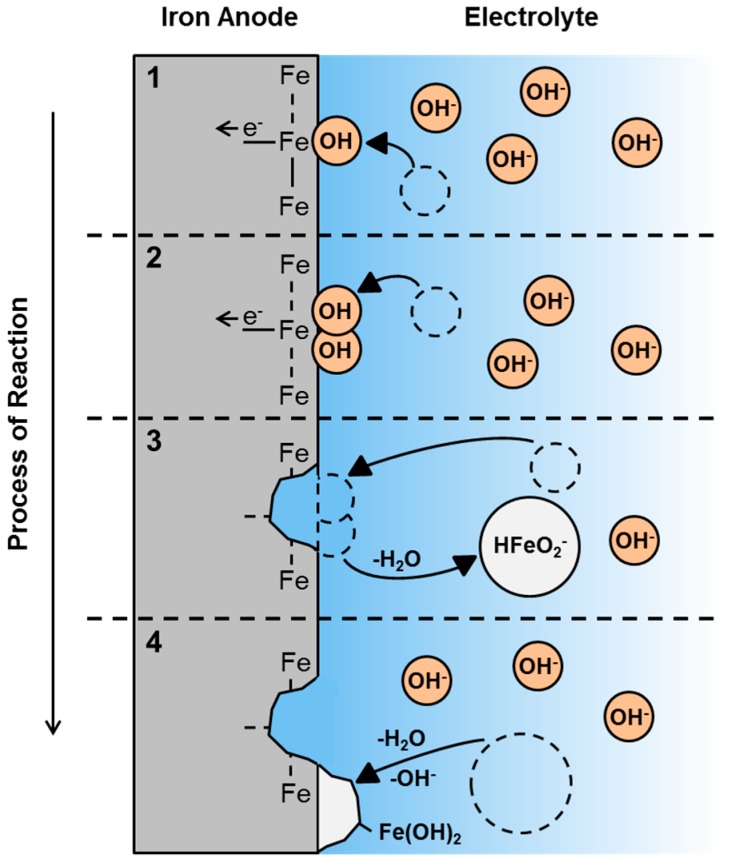
Schematic representation for the first oxidation reaction mechanism of iron in alkaline electrolyte according to Kabanov et al. [234].

The oxidation of Fe to Fe(OH)_2_ is the primary discharge reaction for iron in alkaline Fe-air batteries [222,231]. However, although being investigated for almost a century, the exact process of reaction is still under discussion [209]. The most widely accepted mechanism was proposed by Kabanov et al. in 1947 [234] and was, since then, reconsidered by many researchers such as Dražić et al. [209,235,236]. According to the proposed mechanism, which is schematically depicted in Figure 23, the first oxidation reaction of iron in concentrated alkaline electrolyte involves four distinct reaction steps (I–IV) including the adsorption of two hydroxide anions on the iron electrode surface:

I:       Fe + OH^−^ ⇌ FeOH_ads_ + e^−^   (19)

II:     FeOH_ads_ + OH^−^ ⇌ Fe(OH)_2,ads_ + e^−^ (20)

the dissolution of HFeO_2_^−^ as an intermediate reaction product:

III:    Fe(OH)_2,ads_ + OH^−^ ⇌ HFeO_2_^−^ + H_2_O (21)

and the precipitation of Fe(OH)_2_ on the iron electrode surface due to the limited solubility of HFeO_2_^−^ in concentrated alkaline solution [237]:

IV:      HFeO_2_^−^ + H_2_O ⇌ Fe(OH)_2_ + OH^−^ (22)

As a result of the discharge reaction, it is believed that Fe(OH)_2_ accumulates on the electrode surface until the metallic iron is completely covered by a passivating layer that prevents further reactions of the iron surface beneath the passive film [194,212]. However, while the overall passivating behavior of iron is known for a long time and the existence of the dissolved species has been proven via rotating ring disc experiments by Armstrong et al. in 1971 already [214,238], surprisingly little is known about the microstructural mechanism of the iron hydroxide-formation. As a matter of fact, in the past, the electrochemical oxidation has mostly been investigated either under mild alkaline conditions, e.g., in borate buffer (pH = 8.4) [210,239,240], or via ex-situ techniques, which do not necessarily apply to iron anodes in concentrated alkaline Fe-air batteries [241]. Considering the Pourbaix diagram in Figure 19, it is clear that the conditions at pH = 8.4 differ significantly from the conditions at pH = 12 and higher [193]. Moreover, from fundamental considerations it is known that the passive film on iron is subject to non-negligible changes depending of the environment, i.e., depending on the presence of airborne oxygen and water [203,241]. Thus, in-situ investigations in concentrated alkaline media are mandatory aiming at an in-depth understanding of the microstructure and the composition of the passivating layer, but have rarely been reported in literature due to the challenging measurement conditions [221].

Tackling the surface layer-formation on iron in concentrated alkaline electrolyte, until today, only very few in-situ investigations have been reported, although they are very important for battery research and corrosion science [241]. One of the first in-situ investigations that has been published so far was performed by Geronov et al. using Mössbauer spectroscopy in 5M KOH, identifying Fe(OH)_2_ and *β*-FeOOH as the main discharge products of the first and the second discharge plateau, respectively [231]. The latter is in excellent agreement with subsequent findings by Neugebauer et al., who investigated the electrochemical reactions of iron in 5M KOH via in-situ ATR-FTIR spectroscopy observing the formation of Fe(OH)_2_ and β-FeOOH as well [242]. Huang et al. investigated the oxidation of iron via in-situ optical ellipsometry, discovering evidence for a bilayer structure of the passive film consisting of Fe_3_O_4_ on the inside and α-FeOOH rather than Fe_2_O_3_ on the outside after repeated galvanostatic charge-/discharge between Fe and Fe(III) in 1M NaOH [240]. Furthermore, Schmuki et al. investigated the surface layer formation by in-situ X-Ray absorption near edge spectroscopy and a laser reflection technique and observed that the passivating layer alternates in thickness depending on the polarization of the iron electrode in 0.1M NaOH [206].

Beyond spectroscopy techniques, the passivation of iron has also been investigated via scanning probe microscopy (SPM), providing detailed topography images of the electrode surface during electrochemical cycling [205,221]. Using atomic force microscopy (AFM) in 1M NaOH, Müller-Zülow et al. surprisingly observed the formation and growth of comparatively large polyhedral surface particles rather than the formation of a homogenous surface layer that has been reported for mild alkaline conditions [205,243]. The results by Müller-Zülow et al. are especially consistent with the observations by Oelkrug et al., who investigated the topography evolution of iron in 1M NaOH via in-situ angular-resolved scattering of coherent laser light. In this investigation, Oelkrug and co-workers observed the evolution of surface crystallites, which were fixed on individual spots on the iron electrode and evolved during repeated charge-/discharge of the anode [204]. Moreover, in a recent in-situ EC-AFM investigation, the surface particle growth in concentrated alkaline electrolyte was found to proceed locally on preferential precipitation sides while also being continuous during both oxidation and reduction of the iron electrode. The latter provides a unique insight into the passivation behavior of iron, which led to the proposition of a phenomenological model for the topography evolution of iron in concentrated alkaline electrolyte that can explain the capacity increase of Fe-air batteries at the beginning of the electrochemical cycling [221].

### 3.6. Electrode Concepts

**Figure 24:**
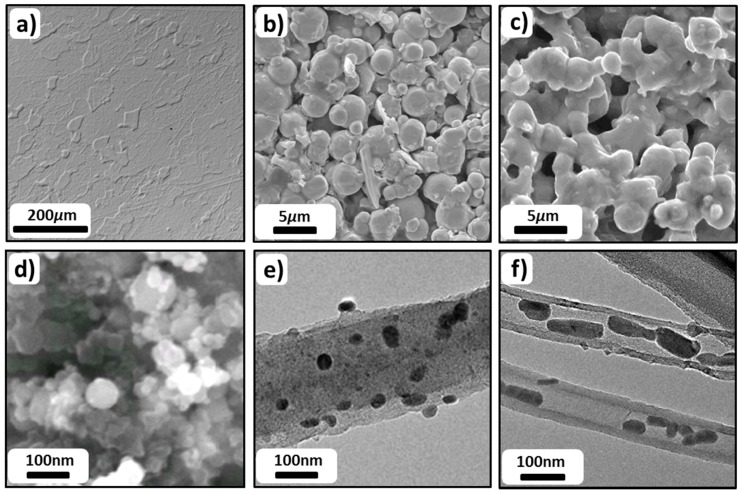
Electrode concepts for Fe-based- and Fe-air batteries. (**a**) Plane iron electrode sheets suitable for fundamental analyzes (Reprinted with permission from *Nano Energy*, 41, 706–716 (2017). ©2017, Elsevier [221]. (**b**) Pressed-plate iron electrode consisting of carbonyl iron powder, performance-enhancing additives and binder. (**c**) Sintered iron electrode prepared from pressed carbonyl iron powder (Reprinted with permission from *J. Electrochem. Soc.*, 164, A418–A429 (2017). ©2017, The Electrochemical Society (ECS)) [100]. (**d**) Nanoparticulate iron electrode consisting of precipitated ferrous material on carbon prepared from dissolved FeCl_2_ as a precursor (Reprinted with permission from *J. Electrochem. Soc.*, 164, A1148–A1157 (2017). ©2017, ECS) [96]. (**e**) Nanoparticulate Fe_2_O_3_-loaded carbon nanofibers (Reprinted with permission from *J. Electrochem. Soc.*, 160, A1442–A1445 (2013). ©2013, ECS) [228]. (**f**) Fe_2_O_3_-filled carbon nanotubes as a negative electrode for Fe-air batteries (Reprinted with permission from *J. Power Sources*,** 178, 393-401 (2008). ©2008, Elsevier) [230].

Research on ferrous anodes for Fe-based- and Fe-air batteries has a comparatively long history dating back to the beginning of the 1970′s and even slightly before, which was nicely summarized in the review about Fe-air batteries by McKerracher et al. [119]. During this long period of time, but especially over the past 10–15 years, there have been several attempts aiming at the improvement of the electrode performance altering the structure, the composition or the processing procedure of the investigated anodes. Figure 24 and Table 5 summarize this development, grouping the individual approaches according to the five most general electrode concepts: plane electrode sheets, pressed-plate microparticles, sintered electrodes, nanoparticles and nanoparticle-loaded carbon structures.

#### 3.6.1. Plane Electrode Sheets

Starting with the simplest approach, Figure 24a shows a laser scanning microscope image of a polished polycrystalline iron electrode sheet, which has effectively been employed for a fundamental investigations of surface reaction processes on iron in concentrated alkaline electrolyte [221]. However, while the approach of an almost entirely flat surface is excellent for fundamental research, its practical applicability is limited due to the comparatively small surface area. During the experiments with this kind of electrode, a maximum reversible discharge capacity of only 6.8 µAh/cm^2^ in 0.5M KOH was achieved (cf. Table 5) [221].

#### 3.6.2. Pressed-Plate Microparticles

The SEM-image in Figure 24b depicts one of the most common approaches for the realization of high performance iron anodes, which has originally been proposed by Manohar et al. and was investigated by many researchers afterwards [173,174,177,184]. For this type of porous iron electrode, all types of (commercially) available (carbonyl) iron or iron oxide powder [245] are pressed onto a current collector using a polymer binder and bismuth sulfide as performance-enhancing additive. Depending on the actual composition and the charging conditions, a discharge capacity of up to 400 mAh/g_Fe_ (first plateau) and a reversibility of more than 1200 charge-/discharge cycles (100%-DoD) has been reported for this type of electrode, which is a remarkable performance considering the ease of preparation, cost and availability of the electrode material (cf. Table 5) [109,177,221]. Moreover, the underlying concept is also flexible in terms of electrode composition, which makes pressed-plate (carbonyl) iron electrodes very attractive for industrial energy storage applications [43].

#### 3.6.3. Sintered Iron Electrodes

Figure 24c shows the electrode structure of a sintered carbonyl iron electrode, which has recently been reported by Yang et al. [100], indirectly pursuing the fundamental research by Öjefors et al. [190,222,237,246]. At a first glance, the sintered iron electrode appears to be almost identical compared to the pressed-plate carbonyl iron electrode reported by Manohar et al. However, the electrode was sintered for 15 min at 850 °C in argon atmosphere, which provides a higher mechanical stability of the anode given the interconnected electrode structure. The sintered iron electrodes realized by Yang and co-workers delivered an excellent coulombic efficiency as well as the highest cycle life of an iron electrode reported in literature so far (cf. Table 5), even though electrode additives have not been employed yet. Leaving the slightly more complicated preparation procedure aside, sintered carbonyl iron electrodes are capable of 3500 charge-/discharge cycles (100%-DoD) at a stable discharge capacity of 192 mAh/g_Fe_ at 1C (1C: j = 200 mA/g_Fe_) and a coulombic efficiency of 97%. The latter marks an outstanding electrode performance with respect to long term stability, which has only to be improved regarding the overall discharge capacity to be practically applicable (80 wt.-% of electrode material still unused). Practical applicability might be achieved by utilizing electrolyte additives, which were reported to be very effective for sintered electrodes [247] or by applying higher charge capacities, which have recently been identified to be a limiting factor for the discharge capacity as well [174].

#### 3.6.4. Nanoparticles

Motivated by the surface area-dependent performance, especially over the past 10–15 years, several researchers investigated the application of nanoparticles for the implementation of high performance rechargeable ferrous electrodes. However, given the increasing reactivity of non-precious nanoparticles, the preparation of iron nanoparticles typically results in the formation of iron oxides, which exhibit unsuitable electrical conductivity, hindering their direct application in batteries. Inferior electrical conductivity of bare iron oxides increases the overpotential for the initial electrochemical formation and the recharge of the individual electrodes, which is detrimental for a highly efficient battery system [168,182,248,249]. Tackling this issue, most researchers addressed the electrical conductivity of iron oxide (nano)-particles by the addition of conductive materials such as carbon black [180,250,251], nickel powder [252], nickel foam [227] and other electrically conductive additives [211] or by the preparation of sophisticated Fe/C-composite materials [96,175,185,244]. In a recent publication, Figueredo-Rodriguez et al., for example, reported the utilization of Fe_2_O_3_/C-composite electrodes for the use in Fe-air full-cells, which provided an excellent discharge performance of about 400 mAh/g_Fe_ (first discharge plateau) at a discharge current density of 10 mA/cm^2^ for more than 20 deep-discharge cycles [96]. The corresponding iron electrodes, depicted in Figure 24d, consisted of nanocrystalline material with a particle size of 20–50 nm and Bi_2_S_3_ as a performance-enhancing additive, which was ballmilled together with the basic material. The original procedure behind this preparation method was first described by Hang et al. in 2005, who investigated electrochemical properties of Fe_2_O_3_-loaded carbon electrodes [175]. Afterwards, similar materials were also applied by Kitamura et al., who analyzed the impact of different binders on the performance of Fe_2_O_3_/C-composite electrodes, which yielded a reversible capacity of up to 700 mAh/g_Fe2O3_ (first & second plateau) at a discharge current density of 5 mA/cm^2^ depending on the actual type of binder [183].

Beyond co-milling of carbon and iron oxide nanoparticles, another excellent preparation approach for the realization of carbon-supported iron oxide materials was reported by Sundar Rajan et al., who investigated thermal co-decomposition of ferrous oxalate dihydrate in the presence of varying amounts of polyvinyl alcohol (PVA) in order to prepare in-situ carbon grafted electrode materials. As a result of the co-decomposition process, they found a remarkable iron electrode material, which rendered a specific discharge capacity in excess of 400 mAh/g_Fe_ (first plateau only) with a faradaic efficiency of 80% that can be discharged both at high and low rates [244].

#### 3.6.5. Nanoparticle-loaded Carbon Structures

Besides the application of Fe/C-composite materials, Hang et al. also reported the investigation of iron oxide-loaded carbon electrode structures [172,228,230]. With respect to this, they particularly investigated the electrochemical properties of nanosized Fe_2_O_3_-loaded carbon nanofibres [228] and nanosized Fe_2_O_3_-filled carbon nanotubes [230] (Figure 24e–f), which are not only interesting from a materials design but also from a performance point of view. Both types of electrodes exhibited excellent electrochemical properties, particularly in terms of the individual discharge performance. The nanosized Fe_2_O_3_-filled carbon nanotubes delivered a discharge capacity of 500 mAh/g at a discharge current density of up to 0.2 mA/cm^2^ for more than 30 cycles and the Fe_2_O_3_-loaded carbon nanofibres provided a discharge capacity of 550 mAh/g at a discharge current density of 2.0 mA/cm^2^ for more than 50 cycles.

Following the fundamental research by Hang et al., over the past five years, several research groups applied the idea of carbon structure-supported iron oxide materials as well [95,253,254]. In this regard, two particularly outstanding results were reported by Wang et al. and Shangguan et al., who made use of graphene nanosubstrates in order to prepare high performance electrodes. Doing so, Wang et al. achieved an FeO_x_/graphene hybrid material, which is capable to provide a discharge capacity of up to 377 mAh/g at an oxidation scan rate of 5 mV/s. Furthermore, combined with a high performance Ni(OH)_2_/MultiWall Nanotube (MWNT) cathode, it is even possible to operate an ultrafast nickel-iron rechargeable battery with a maximum specific discharge current of 37 A/g and a specific discharge capacity of up to 126 mAh/g, which calculates back to a specific power of 15 kW/kg and a specific energy of 120 Wh/kg [95].

Similar to Wang et al., Shangguan et al. applied graphene as a substrate for the application in Fe-based batteries as well. However, instead of using iron or iron oxide nanoparticles, they directly applied FeS anchored on graphene oxide nanosheets, combining active material and performance-enhancing additive to a single battery material. As a final result of their work, Shangguan and co-workers were able to perform more than 300 charge-/discharge cycles with a capacity above 300 mAh/g at 2C, while the electrode was capable to deliver a discharge capacity of up to 250 mAh/g at 20C, which is equal to a discharge current density of 6 A/g [253].

#### 3.6.6. Summarizing Remarks

Evaluating the research mentioned in the previous paragraphs, Table 6 compares strengths and weaknesses of the individual electrode concepts regarding the available surface area, mechanical stability, electrical conductivity, the applicability of electrode additives, carbon content and the electrochemical performance. Starting with the “Plane Electrode Sheets”, it is clear that its electrochemical performance is limited due to the very low surface area. Furthermore, the simplest electrode concept does not offer the possibility to apply electrode additives, which might enhance the electrode capabilities. However, the mechanical stability, given the continuous solid-state bonds and the flat surface is an advantage for fundamental research. In contrast to that, “Pressed-Plate Microparticle”-electrodes rely on the cohesion of its constituting particles, which has occasionally to be ensured by the addition of binder. In return for the application of particles, a much larger surface area as well as the applicability of electrode additives is obtained. The latter guarantees for a certain, charge-/discharge capacity dependent electrochemical performance. Beyond that, the electrochemical performance may only further be increased by sintering, since an extended electrochemical cyclability for more than 3500 cycles (100% DoD) has only been reported by Yang et al. [100]. However, sintering also diminishes the possibilities to apply electrode additives, since the employed additives might not be stable under the required sintering conditions.

Omitting heat treatments, descent electrical conductivity may be found on nanoparticulate electrodes once the initial electrode material (iron oxide) has been reduced and forms conductive pathways through the electrode with the help of carbon or other conductive additives. However, the application of nanosized, but only weakly bonded particles also bears the risk of rapid mechanical degradation due to gradual particle detachment from the electrode. Accordingly, it can be concluded that there is not just one electrode concept that satisfies all application requirements. In fact, each concept has its pros and cons, which have to be considered for the individual application.

### 3.7. Electrode- and Electrolyte Additives

The electrochemical performance of rechargeable anodes for Fe-air batteries largely depends on the reversibility of the resulting discharge products and the extent of the HER in aqueous electrolyte during the recharge of the battery. In order to improve the battery performance, in parallel to the investigation of novel electrode concepts, many researchers investigated the impact of additives on the performance of ferrous electrodes [109,186,190,255,256]. Doing so, over the course of several decades, many additives have been identified to enhance the cyclability of the investigated electrodes due to the following objectives [186]: First, enhancement of the Fe/Fe(OH)_2_ reaction rate according to Equation (10). Second, increase of the electrical conductivity of the ferrous-anode, particularly in the discharged state. Third, modification of the electrode morphology. Fourth, increase of the overpotential for the HER [186,256].

Depending on the chemical nature of the individual additives, two groups of materials can easily be distinguished according to the site of application: electrode- and electrolyte additives. Regarding electrode additives, it has recently been shown by Posada et al. and Manohar et al. that several solid materials may directly be incorporated into the porous structure of pressed-plate carbonyl iron electrodes, offering the opportunity to optimize the electrode properties with respect to the electrode performance [184,218,236]. Among the different chemicals, currently, the utilization of 4–10 wt.-% Bi_2_S_3_ is favored in order to improve porous iron electrodes by its twofold working principle. First, the decomposition of Bi_2_S_3_ according to Equation (23) leads to the formation of elemental bismuth, which increases the overpotential for the HER [109]. Second, the simultaneous release of sulfide ions enables the formation of electrically conductive FeS according to Equation (24), which is incorporated in the passive discharge layer and improves the reversibility of Fe(OH)_2_ upon recharge:

Bismuth formation:           Bi_2_S_3_ + 6e^−^ ⇌ 2Bi + 3S^2−^       E^0^ = −0.82 V (23)

Iron sulfide formation:        S^2−^ + Fe(OH)_2_ ⇌ FeS + 2OH^−^             (24)

However, the use of solid sulfurous electrode additives is not limited to Bi_2_S_3_ only. Shangguan et al. for example, recently showed the successful application of sublimed sulfur powders as novel anode additives for porous iron electrodes [257].

Furthermore, the use of water-soluble solid chemicals like potassium carbonate and potassium sulfide has been reported to improve the performance of porous iron electrodes as well. Given their water-solubility, K_2_CO_3_ and K_2_S have been used to increase the porosity of pressed-plate iron electrodes, which has a pronounced effect on the cyclability of the anode. Especially for pressed carbonyl iron electrodes using 5–10 wt.% of polymer binder, an explicit tendency to prolonged electrochemical formation due to unfavorable wetting of the inner electrode volume has been reported, which should clearly be omitted by the electrode structure [173,177,218,236].

Regarding electrolyte additives, small amounts of soluble chemicals such as LiOH [255], K_2_S [189], or Na_2_S [182] were employed to improve the performance of ferrous iron electrodes as well. However, although dissolved in the electrolyte, the use of K_2_S, Na_2_S and other sulfide salts does not alter the working principle of sulfides too much. The only difference with respect to the effects of solid Bi_2_S_3_ might be that the dissolved sulfide ions are readily available right from the beginning of the galvanostatic cycling, which might be an advantage with respect to the electrochemical formation. Beyond sulfides, typically, only LiOH poses an additional mode of action for the improvement of the reversibility of iron in alkaline electrolyte. The latter might consist in an altered structure of the passivating layer due to the incorporation of Li, which has already been investigated as early as 1973 [258]. Furthermore, in 2017, it has been shown by Chamoun et al. that the performance of iron electrodes may also be enhanced by the application of potassium stannate in concentrated alkaline electrolyte, which might be due to the alloy formation of iron and tin upon repeated electrochemical cycling [256]. This observation has further been discussed by Paulraj et al., who used in operando charge efficiency measurement to study copper/tin-doped nano-iron electrodes [259].

### 3.8. Electrochemical Formation of Porous Iron Electrodes

**Figure 25:**
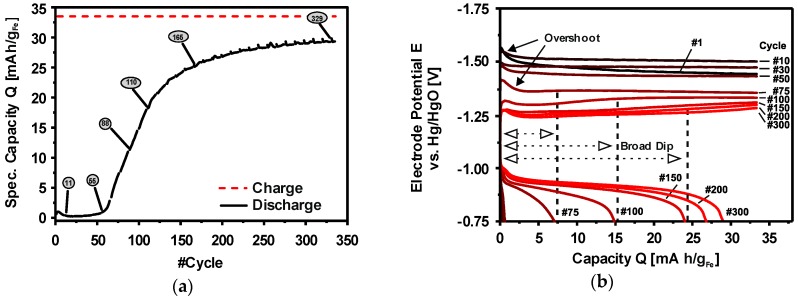
Electrochemical formation of a pores iron electrode in 6M KOH. (**a**) Formation curve for a pressed-plate carbonyl iron electrode over the course of about 350 repeated charge-/discharge cycles (100% DoD). (**b**) Charge-/discharge curves corresponding to the graph in (a). (Reprinted with permission from *J. Appl. Electrochem.*, 48, 451–462 (2018). ©2018, Springer) [174].

Unlike the intercalation chemistry of lithium-ion batteries, the electrochemistry of iron in alkaline electrolyte is confined to surface reactions, which is crucial for non-prenanostructured electrodes. Due to the surface reaction confinement, the application of porous or particulate electrodes is required to guarantee for a practically relevant electrode performance [119]. In this regard, particularly the use of pressed-plate microparticles has proven to be a versatile approach to create rechargeable iron electrodes. In addition to a much higher surface area, pressed-plate iron electrodes offer the chance to design the electrode structure as well as the actual electrode composition [93,189,236]. However, pressed-plate electrodes also exhibit a pronounced tendency to electrochemical formation, i.e., a significantly increasing discharge capacity of the iron-anode in the beginning of the electrochemical cycling that may last for several tens of cycles depending on the investigated electrode [174,223,236]. Taking the electrochemical formation into account, Figure 25 depicts the formation behavior of a porous, carbonyl iron electrode in 6M KOH. Due to the repeated charge-/discharge (100% DoD) the discharge capacity of the electrodes increases after an initial delay and finally merges into a stable plateau depending on the applied charge capacity. Given the oxidation mechanism of iron in alkaline electrode, the capacity increase during the formation is explained by an increasing active surface area of each (carbonyl) iron particle and an increasing number of particles contributing to the electrochemical reaction of the electrode [236,260]. Furthermore, the eventually stable discharge capacity of the electrode is governed by an equilibrium between the loss of active surface area due to the accumulation of discharge residuals and the gain in surface area due to particle coarsening as well as the increasing number of active particles [174].

## 4. Conclusions

Silicon and iron are two of the most abundant elements in the Earth’s crust, which makes these two excellent choices for the implementation of resource-efficient and potentially cheap batteries. In this review, the electrochemistry, reaction mechanisms, electrode design and performance of silicon- and iron electrodes have been discussed with respect to the application as anode materials in metal-air batteries. The standard electrode potentials for the redox reactions of silicon (−1.69 V) and iron (−0.88 V) differ by 0.81 V and both elements are subject to the formation of thin surface oxide layers at ambient conditions. Nevertheless, when operated in concentrated alkaline electrolytes, the redox reaction mechanisms and the reactions concurrent to the redox reactions are substantially different.

Upon reduction, the competing reaction for the recharge of silicon and iron in alkaline electrolyte is the hydrogen evolution reaction due to water electrolysis. In case of formed iron electrodes and a careful choice of the operating parameters, this side reaction can be kept at a moderate level. In contrast to that, high overpotentials in addition to the higher standard electrode potential currently prevent the realization of an electrochemical reduction of silicon electrodes in alkaline electrolytes completely. Therefore, the application of silicon in metal-air batteries is limited to primary use so far, whereas iron-air batteries can be operated as secondary systems.

Upon oxidation, the discharge reaction of iron occurs via a dissolution and precipitation mechanism. However, the solubility of the intermediate ionic species in alkaline electrolytes is very low, which results in the formation of a passivating layer on the iron surface, so reactions are limited to a thin surface layer. In contrast to that, the oxidation of silicon in alkaline electrolytes leads to soluble silicates, which allow a continuous discharge until the silicon anode has been consumed completely. However, non-passivated silicon surfaces are subject to substantial corrosion. Furthermore, for both materials, only low discharge currents can be realized on planar surfaces.

In case of iron anodes, the limits of the surface reaction confined performance can be overcome by providing higher surface areas in form of porous and/or nanoparticulate electrodes, which may be subject to nanostructuring by electrochemical formation. The optimization of the electrode composition by the addition of electrode additives, the morphology and the electrochemical conditions for the formation process are major subjects of current research.

In case of silicon anodes, strong hydrogen evolution as a result of the corrosion reaction concomitant to the discharge reaction largely prevents a straightforward enhancement of the electrode design by higher surface areas. Precondition for the application of porous or nanoparticulate electrodes is the suppression of the hydrogen formation. Approaches thereto are the development of corrosion inhibitors, enhanced specification of the silicon materials with respect to the dopant content and the application of ionic liquids as an alternative to alkaline electrolytes.

Current status for the performance of iron-air batteries is the implementation of reversible cycling for a high number of cycles with specific energies between 300 and 500 Wh/kg. Silicon-air primary batteries can provide specific energies of up to 1660 Wh/kg in ionic liquid and up to 140 Wh/kg in alkaline electrolyte. At present, for both types of batteries the specific power is lower than for Li-ion batteries. However, main advantage of alkaline iron- and silicon-air batteries is their excellent resource-efficiency. With respect to future developments, the high theoretical specific energies provide a wide range for the improvement of silicon- and iron-air batteries.

## Figures and Tables

**Table 1:** Theoretical energies of various metal-air battery anode materials. (Asterisk and dagger indicate the corresponding type of electrolyte, i.e., aqueous (*) or non-aqueous electrolyte (✝); ’⇌’, ‘(⇌)’ and ‘→’ indicate reversible, limitedly reversible and primary metal-air batteries (MAB) systems; column “n” provides the number of transferred electrons during the electrochemical reactions. Molar masses and mass densities obtained from [42].

System	Primary Reaction	n	Full Cell Voltage	Mass Density (Metal)	Specific Energy (Metal)	Energy Density (Metal)	Mass Density (Dis. Product)	Specific Energy	Energy Density	Ref.
			––––––––––––––– *Excluding Oxygen Uptake* –––––––––––––––				–––––– *Including Oxygen Uptake* –––––			
			[V]	[kg/L]	[Wh/kg]	[Wh/L]	[kg/L]	[Wh/kg]	[Wh/L]	
**Fe-air**	Fe ⇌ Fe(OH)_2_	2 *	1.28	7.87	1229	9677	3.40	764	2598	[43]
**Zn-air**	Zn ⇌ ZnO	2 *	1.65	7.14	1352	9653	5.61	1086	6092	[44]
**K-O_2_**	K → KO_2_	1 ^✝^	2.48	0.89	1700	1513	2.16	935	2019	[45]
**Na-O_2_**	Na → Na_2_O_2_	1 ^✝^	2.33	0.97	2716	2634	2.81	1601	4409	[46]
	Na → NaO_2_	1 ^✝^	2.27		2646	2567	2.20	1106	2433	[47]
**Ca-O_2_**	Ca → CaO_2_	2 ^✝^	3.38	1.54	4520	6961	2.9	2516	7296	[48,49]
	Ca → CaO	2 ^✝^	3.13		4186	6446	3.34	2996	10007	
**Mg-Air**	Mg → Mg(OH)_2_	2 *	2.77	1.74	6098	10610	2.37	2848	6750	[50]
**Mg-O_2_**	Mg → MgO	2 ^✝^	2.95		6493	11299	3.6	3919	14108	[51]
**Si-air**	Si →Si(OH)_4_	4 *	2.09	2.33	8001	18644	~1.8	2334	4201	[52]
	Si → SiO_2_	4 ^✝^	2.21		8461	19748	2.19	3947	8643	[53]
**Al-air**	Al →Al(OH)_3_	3 *	2.71	2.70	8091	21837	2.42	2784	6737	[54]
**Al-O_2_**	Al (⇌) Al_2_O_3_	3 ^✝^	~2.1		~6258	~16897	3.97	3311	13145	[55]
**Li-O_2_**	Li (⇌) Li_2_O_2_	1 ^✝^	2.96	0.53	11430	6104	2.31	3458	7988	[40,41]
**Li-O_2_**	Li (⇌) Li_2_O	1 ^✝^	2.91		11238	6001	2.01	5220	10492	

**Table 2:** Theoretical values and experimental results for the performance of recent Me-air- (Me-O_2_-) batteries and half cells reported in literature. (Specific energies are given based on the weight of the metallic electrode only; C—abbreviates carbon; Cath.—abbreviates cathode; experimental conditions differ significantly depending on the investigated MAB-system).

System	Discharge Product	Full Cell Voltage	Specific Energy	Energy Density	Reported Specific Energy			Reported Reversibility	
		––––––––––––––– *Theoretical Values* –––––––––––––––			––––––––––––––– *Experimental Results* –––––––––––––––				
					Performance	Condition	Ref.	Cycles	Ref.
		[V]	[Wh/kg]	[Wh/L]					
**Fe-air**	Fe(OH)_2_	1.28	1229	9677	453 Wh/kg_Fe_	[b,c,d,e]	[95]	3500 ^[b,d]^	[100]
**Zn-air**	ZnO	1.65	1352	9653	>700 Wh/kg_Zn_	[a,c,d]	[94]	>75 ^[a,c]^	[97]
**K-O_2_**	KO_2_	2.48	1700	1513	~19,500 Wh/kg_C_	[a,c,d]	[101]	>200 ^[a,c]^	[101]
**Na-O_2_**	Na_2_O_2_	2.33	2716	2634	~18,300 Wh/kg_C_	[a,c,d]	[46,47]	>20 ^[a,c]^	[102]
	NaO_2_	2.27	2646	2567					
**Ca-O_2_**	CaO_2_	3.38	4520	6961	tbd	-	-	tbd	[44,45]
	CaO	3.13	4186	6446					
**Mg-Air**	Mg(OH)_2_	2.77	6098	10610	~2750 Wh/kg_Cath._	[a,c,d,f]	[103,104]	<10 ^[a,c,d]^	[51]
**Mg-O_2_**	MgO	2.95	6493	11299					
**Si-air**	Si(OH)_4_	2.09	8001	18644	~1600 Wh/kg_Si_	[a,c,d]	[52]	not yet	[50]
	SiO_2_	2.21	8461	19748					
**Al-air**	Al(OH)_3_	2.71	8091	21837	~2300 Wh/kg_Al_	[a,c,d]	[55]	limited	[92,100]
**Al-O_2_**	Al_2_O_3_	~2.1	~6258	~16897					
**Li-O_2_**	Li_2_O_2_	2.96	11430	6104	>11,000 Wh/kg_C_	[a,c,d]	[41,105]	>250 ^[a,c]^	[98]
**Li-O_2_**	Li_2_O	2.91	11238	6001					

Conditions: a—anode sheet/foil; b—porous/particulate anode; c—full-cell measurements; d—100% deep discharge; e—repeated charge-/discharge; f—elevated temperature.

**Table 3:** Overview over the possible Si-air battery systems.

Types	Electrolyte	Cell Reactions	Remarks	Ref.
**Non-Aqueous**	EMIm(HF)_2.3_F	Si + 12(HF)_2_F^−^ ⇄ SiF_4_ + 8(HF)_3_F^−^ + 4e^−^ O_2_ + 12(HF)_3_F^−^ + 4e^−^ ⇄ 2H_2_O + 16(HF)_2_F^−^ SiF_4_ + 2H_2_O + 4(HF)_2_F^−^ ⇄ SiO_2_ + 4(HF)_3_F^−^ Si + O_2_ ⇄ SiO_2_	Higher energy densities and lower corrosion rates compared to aqueous systems. Discharge limitation mainly by Si anode as well as pore clogging and catalyst conversion problems in the air cathode. Environmental concerns due to fluoride content and high cost. No rechargeability.	[53,123,142,143,144]
**Aqueous**	KOH	Si + 4OH^−^ ⇄ Si(OH)_4_ + 4e^−^ Si(OH)_4_ + 2OH^−^ → SiO_2_(OH)_2_^2−^ + 2H_2_O O_2_ + 2H_2_O + 4e^−^⇄4OH^−^ Si + 2OH^−^ + 2H_2_O → SiO_2_(OH)_2_^2−^ + 2H_2_	Cost effective, environmentally friendly, easy handling, high ionic conductivity. Higher corrosion rates, low mass conversion efficiencies, and no rechargeability.	[127,128]
**Solid State**	Stabilized zirconia (CSZ)	2O^2−^ ⇄ O_2_ + 4e^−^ 2Si + O_2_ ⇄ 2SiO Si + O_2_ ⇄ SiO_2_ O_2_ + 4e^−^ ⇄ 2O^2−^	Rechargeability with round-trip efficiency of 45%. Full active mass consumption of Si anode. High working temperature, low energy density	[139]
	Gel polymer electrolyte (EMIm(HF)_2.3_F)	See non-aqueous	Mechanically strong and flexible GPE, extended discharge times, no drying out. Lower ionic conductivity, lower discharge voltages.	[140]

**Table 4:** Comparison of the discharge performances by means of discharge voltage, corrosion rate, mass conversion efficiency, and specific energy for different type of Si wafers after discharging with 100 µA/cm^2^ at 25 °C [86].

Si Wafer Type	Discharge Voltage [V]	Corrosion Rate [nm/min]	Mass Conversion Efficiency [%]	Specific Energy [Wh/kg]
As<100>	1.05	3.6	30.5	1350.2
As<111>	0.86	2.0	43.6	1660.5
Sb<100>	0.98	3.7	29.7	1230.3
Sb<111>	0.81	2.3	40.6	1430.6
B<100>	0.69	1.9	44.3	1340.1
B<111>	0.66	1.8	47.2	1370.8

**Table 5:** Composition and performance of different iron electrode concepts. (Please note: The lower five entries consider both first and second (deep) discharge reaction of iron, while the other results consider the first discharge reaction only.).

Electrode Concept	Material (size)	Precursor	Preparation	Current Collector	Additives (wt.-%)	Formation	Dis. Capacity (Chr. Capacity)	Reversibility	Ref.
Sheets	Fe (continuous)	None	Polishing	None	None	Yes	6.8 µAh/cm^2^ (200 µAh/cm^2^)	Full-cell > 8 cycles	[221]
Microparticles	Fe (3–5 µm)	None	Die pressing (after 140 °C)	None	8.5% Bi_2_S_3_; 5% PE	Yes	220 mAh/g_Fe_ (300 mAh/g_Fe_)	>350 cycles	[174]
Microparticles	Fe (3–5 µm)	None	(Hot-) pressed at 140 °C	Nickel grid	10% Bi_2_O_3_; 5% FeS; 10% PE; 10% K_2_CO_3_	Yes	240 mAh/g_Fe_ (500 mAh/g_Fe_)	> 1200 cycles	[109]
Microparticles	Fe (3–5 µm)	None	Coating of current collector & short sintering	Nickel mesh	10% graphite; 1% Bi_2_S_3_; 6% PTFE; 0.5% NiSO_4_·7H_2_O	Yes	400 mAh/g_Fe_ (500 mAh/g_Fe_)	>200 cycles	[177]
Microparticles	Fe (<10 µm)	None	Coating of current collector	Nickel foam	11% FeS; 6% PTFE; LiOH; K_2_S	Yes	230 mAh/g_Fe_ (550 mAh/g_Fe_)	>50 cycles	[233]
Sintered Electrodes	Fe (3–5 µm)	Carbonyl Iron	Sintering in Ar at 850 °C	Nickel mesh	NH_4_HCO_3_; Na_2_S	Yes	192 mAh/g_Fe_ (200 mAh/g_Fe_)	>3500 cycles	[100]
Nanoparticles	Fe/Fe_3_O_4_ (not specified)	α-FeC_2_O_4_ 2H_2_O–PVA composite	Combustion of precur. & coating of current collector	Nickel mesh	10% carbon; 1% Bi_2_S_3_; 6% PTFE; 0.5% NiSO_4_·7H_2_O	No	400 mAh/g (500 mAh/g)	>100 cycles	[244]
Nanoparticles	Fe_2_O_3_ (15–50 nm)	FeCl_2_	Hot pressed at 200 °C	Two steel meshes	10% carbon; 4% Bi_2_S_3_; 5% PTFE	No	400 mAh/g (900 mAh/g)	Full-cell >20 cycles	[96]
Nanoparticles	Fe_2_O_3_ (<50 nm)	None	Loaded nickel foam	None	Carbon; 10% binder	Yes	<700 mAh/g (1007 mAh/g)	>100 cycles	[230]
Nanoparticles on Carbon Structures	Fe_2_O_3_ (<50 nm)	Fe(NO_3_)_3_	Rolling of Fe_2_O_3_–filled CNT	None	10% PTFE	No	<500 mAh/g (962 mAh/g)	>30 cycles	[172]
Nanoparticles on Carbon Structures	Fe_2_O_3_ (<20 nm)	Fe(NO_3_)_3_	Rolling of Fe_2_O_3_–filled CNF	None	10% PTFE; 2% Bi_2_S_3_	No	<550 mAh/g (1007 mAh/g)	>50 cycles	[53]
Nanoparticles on Carbon Structures	FeO_x_/graphene (<100 nm)	Fe(OAc)_2_	Loaded nickel foam	Nickel foam	Glucose; PTFE	No	<377 mAh/g (not specified)	>300 cycles	[95]
Nanoparticles on Carbon Structures	FeS/graphene oxide (<100 nm)	FeSO_4_·7H_2_O	Co-precipitation	Nickel foam	10% PTFE; 10% carbon black; 3% Bi_2_S_3_; 0.5% NiSO_4_·7H_2_O	No	<325 mAh/g (400 mAh/g)	>300 cycles	[93]

**Table 6:** Evaluation of the electrode properties for the individual electrode concepts mentioned in Section 3.6. (--/none; -/limited; 0/neutral; +/good; ++/excellent or high).

	Plane Electrode Sheet	Pressed-Plate Microparticles	Sintered Electrodes	Nano-Particles	Nanoparticle-Loaded Carbon Structures
Surface Area	-	0/+	+/++	++	++
Mechanical Stability	++	0/+	+	-	-
Electrical Conductivity	+/++	0	+	-/++	-/++
Applicability of Elec. Additives	--	++	-	++	+/++
Carbon Content	--	+	--	++	++
Electrochemical Performance	-	+	++	+	0/+

## Appendix A

**Table A1:** Transition reaction in Figure A1.

# No.	Reaction
1	Fe ⇌ Fe2+ + 2e−
2	FeOH2+ + 2OH− ⇌ Fe(OH)3
3	Fe2+ + 3OH− ⇌ Fe(OH)3 + 1e−
4	Fe2+ + 2OH− ⇌ Fe(OH)2
5	Fe(OH)2 + OH− ⇌ Fe(OH)3 + 1e−
6	Fe + 2OH− ⇌ Fe(OH)2 + 2e−
7	Fe(OH)2 + OH− ⇌ HFeO2− + H2O
8	Fe + 3OH− ⇌ HFeO2− + H2O + 2e−
9	HFeO2− + H2O ⇌ Fe(OH)3 + 1e−
